# Knockout of the mitoribosome rescue factors Ict1 or Mtrfr is viable in zebrafish but not mice: compensatory mechanisms underlying each factor's loss

**DOI:** 10.1002/2211-5463.70054

**Published:** 2025-05-16

**Authors:** Nobukazu Nameki, Chika Tomisawa, Soichiro Hoshino, Hidehiko Shimizu, Masashi Abe, Sho Arai, Kanako Kuwasako, Naoki Asakawa, Yusuke Inoue, Takuro Horii, Izuho Hatada, Masakatsu Watanabe

**Affiliations:** ^1^ Division of Molecular Science, Graduate School of Science and Technology Gunma University Kiryu‐shi Japan; ^2^ Faculty of Pharmacy and Research Institute of Pharmaceutical Sciences Musashino University Nishitokyo‐shi Japan; ^3^ Laboratory of Genome Science, Biosignal Genome Resource Center, Institute for Molecular and Cellular Regulation Gunma University Maebashi Japan; ^4^ Viral Vector Core Gunma University Initiative for Advanced Research (GIAR) Maebashi Japan; ^5^ Graduate School of Frontier Biosciences Osaka University Suita Japan; ^6^ Present address: Cellular and Structural Physiology Institute (CeSPI) Nagoya University Furo‐cho, Chikusa‐ku Nagoya 464‐8601 Japan; ^7^ Present address: Graduate School of Pharmaceutical Sciences Nagoya University Furo‐cho, Chikusa‐ku Nagoya 464‐8601 Japan

**Keywords:** COXPD7, mice, mitochondrial morphology, mitochondrial ribosome rescue, the YSLDK motif, zebrafish

## Abstract

The mitochondrial translation system contains two ribosome rescue factors, ICT1 and MTRFR (C12orf65), which hydrolyze peptidyl‐tRNA in stalled ribosomes. ICT1 also functions as a ribosomal protein of the mitochondrial large ribosomal subunit (mtLSU) in mice and humans, and its deletion is lethal. In contrast, MTRFR does not share this role. Although loss‐of‐function mutations in *MTRFR* have been linked to human mitochondrial diseases, data on this association in other vertebrates are lacking. Here, attempts to generate *Mtrfr* knockout mice were unsuccessful. However, knockout zebrafish lines were successfully generated for both *ict1* and *mtrfr* (*ict1* ^−/−^ and *mtrfr* ^−/−^). Both knockout lines appeared healthy and fertile. *ict1* ^−/−^, *mtrfr* ^−/−^, and wild‐type adult caudal fin cells showed significant differences in mitochondrial morphology. The *ict1* deletion affected the network properties more than the number of individuals and networks, whereas the *mtrfr* deletion exhibited the opposite effect. Additionally, the survival rates of the knockout line larvae were significantly lower than those of the wild‐type larvae under starvation conditions. These results suggest that *ict1* and *mtrfr* are required for survival under specific stress conditions, whereas *ict1* ^−/−^ and *mtrfr* ^−/−^ involve different compensatory mechanisms in response to loss of either factor under nonstress conditions. Ict1 proteins from all teleosts, including zebrafish, lack the N‐terminal mtLSU‐binding motif found in most metazoans, suggesting that Ict1 does not function as a ribosomal protein in teleosts. Thus, Mtrfr may partially compensate for the loss of Ict1. In conclusion, zebrafish appear to exemplify a limited category of vertebrates capable of enduring genetic abnormalities in *ict1* or *mtrfr*.

## Graphical abstract

Mitochondria contain two mitoribosome rescue factors, ICT1 and MTRFR (C12orf65). ICT1 also functions as a mitoribosomal protein in mice and humans, and its loss is lethal. Although *Mtrfr* knockout mice could not be generated, knockout zebrafish lines for *ict1* and *mtrfr* were established. Zebrafish Ict1 lack the N‐terminal mtLSU‐binding (YSLDK) motif; zebrafish represent a limited category of vertebrates capable of tolerating defects in *ict1* or *mtrfr*.

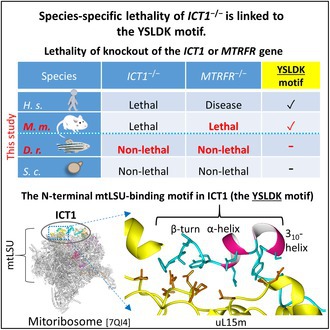

**Abbreviations**
COXPD7: combined oxidative phosphorylation deficiency‐7
CRISPR/Cas9: clustered regularly interspaced palindromic repeat/CRISPR‐associated protein 9
cryo‐EM: cryo‐electron microscopy
dpf: days postfertilization
mtLSU: mitochondrial large ribosomal subunit
OXPHOS: oxidative phosphorylation

Mitochondria have unique transcription and translation systems for the mitochondrial genome or DNA (mtDNA) that encodes a limited number of genes (13 proteins, 2 rRNAs, and 22 tRNAs in humans, mice, and zebrafish) [1, 2]. Translating ribosomes are stalled on mRNA due to various reasons irrespective of species or organelles. A previous study using deep sequencing analysis investigated sources of mRNAs lacking a stop codon (nonstop mRNA) generated from the human mitochondrial genome and revealed that several nonstop mRNAs, with different production processes, occur in healthy cells and tissues [3]. This finding indicates that mitochondrial ribosomes (mitoribosomes) at the 3′ ends of nonstop mRNAs are common. These ribosomes significantly impact the efficiency of ribosome recycling and, consequently, translational efficiency or output (yield), which may ultimately be fatal to cells [4]. In mitochondria, irrespective of eukaryotic species, the specific translation system comprises two mitoribosome rescue factors, ICT1 and C12orf65 (renamed MTRFR, as described later). These factors are homologous since they function as peptidyl‐tRNA hydrolases, with the Gly‐Gly‐Gln (GGQ) motif residues serving as a catalytic site [5, 6, 7, 8]. Following the structured GGQ‐containing domain (GGQ domain), both proteins feature an unstructured C‐terminal extension in a free form, which differs in sequence between the two factors and plays a key role in their respective functions [7, 8]. Notably, the GGQ domains of the rescue factors show homology with one another and with the catalytic domain (domain 3) of the bacterial‐type release factor (RF) (RF1 and RF2 in bacteria; mtRF1a and mtRF in mitochondria). This suggests that ICT1, C12orf65, and the bacterial‐type RFs evolved from a common ancestor and subsequently acquired different roles [9]. The particular functions of ICT1 and C12orf65 elucidated to date, along with the remaining unresolved points, are outlined below.

ICT1 was first identified as a mitoribosome rescue factor [5]. Many bacteria, though not all, contain an ICT1 ortholog known as ArfB (YaeJ) [5, 7, 10, 11, 12]. ICT1/ArfB basically enters the A‐site of a stalled ribosome caused by nonstop mRNA and hydrolyzes peptidyl‐tRNA in the P‐site. This activity allows for the release of the stalled ribosome, facilitating ribosome recycling. According to structural studies of ArfB and ICT1 [13, 14], when ICT1/ArfB binds to a stalled ribosome caused by nonstop mRNA, a helical region is formed by a section of the C‐terminal extension of ICT1/ArfB, which lies in the mRNA entry channel downstream of the A‐site vacant in the 30S subunit. Residues involved in the specific binding to the mRNA entry channel are highly conserved across ICT1 and ArfB proteins from various species [8]. Accordingly, the C‐terminal extension is considered a sensor for discriminating between stalled and actively translating ribosomes. The GGQ domain is positioned in the A‐site, akin to that of RFs, with the GGQ motif residues situated at the peptidyltransferase center of the large ribosomal subunit. This positioning allows the peptidyl‐tRNA at the P‐site to be hydrolyzed.

Of note, ICT1 has a dual function as it also serves as an integral component of the large ribosomal subunit of the mammal mitoribosome (mtLSU), also designated mL62 (MRPL58) based on the nomenclature of ribosomal proteins [5, 15]. The ICT1 protein bound to the mtLSU connects the central protuberance, the components of which are specific to the mitochondria, to the main body of the mitoribosome, contributing to the structural stabilization of mtLSU, and consequently, the entire mitoribosome [16]. A similar dual function has been observed in mitochondrial tRNA^Val^, which also functions as an additional rRNA constituent that is incorporated into the central protuberance [16, 17]. During the rapid evolution or increasing divergence of mitoribosomes, putative structural instabilities, such as those induced by the absence of certain mitochondrial rRNA segments, are often compensated for by pre‐existing elements, a phenomenon known as structural patching [18]. Once ICT1 is bound to a mitoribosome, it cannot be released and will not act as a rescue factor [10]. Whether some free ICT1 is present in mitochondria, whether free ICT1 is produced, for example, by a mechanism that inhibits its binding to a mitoribosome, or whether free ICT1 exists in a cell or tissue‐dependent manner, remains to be determined.

ICT1 is essential for cellular maintenance, cell viability, proliferation, and development. In human cultured cells, depletion of ICT1 using ICT1‐specific siRNA significantly reduced growth compared with control cells, decreasing the levels of mitochondrial protein synthesis for 13 genes [5, 7]. Given the lack of recorded instances of ICT1 mutations linked to diseases, mutations that lead to loss of function in ICT1 would be fatal in humans. A comprehensive study of mitochondrial ribosomal proteins has demonstrated that *Ict1* knockout mice are embryonically lethal [19, 20]. Such severe effects of ICT1 dysfunction may be attributed to a combination of its loss of function as a ribosomal protein and as a rescue factor. Further studies remain warranted to elucidate the details of this combination.

C12orf65, a second rescue factor, began with a report describing that loss‐of‐function mutations in a nuclear gene, *MTRFR*, in two unrelated pedigrees lead to mitochondrial encephalomyopathy [6]. Analysis of fibroblasts from patients showed that a 1‐bp deletion in *MTRFR*, resulting in a premature stop codon, impaired total mitochondrial translation for 13 genes. This caused a significant reduction in the level of oxidative phosphorylation (OXPHOS) complexes I, IV, and V, and a more modest decline in complex III. In contrast to the ICT1‐depleted cells, the patient fibroblasts showed no adverse effects on the steady‐state levels of mitoribosomal proteins or the assembly of mitoribosomal subunits [6, 21]. In cultured cells, siRNA‐mediated knockdown of C12orf65 inhibited cell proliferation, but less than ICT1 knockdown [8]. Several studies have demonstrated that the *MTRFR* gene is subject to various loss‐of‐function mutation patterns associated with disease [22, 23, 24, 25, 26, 27, 28, 29, 30, 31, 32]. If the mutant gene is expressed, the resulting protein is incomplete or dysfunctional, with the length of the protein varying based on the specific mutation [25, 27, 28]. *MTRFR* deficiency is characterized by a wide range of phenotypes based on the mutations; the three primary clinical features are optic atrophy, peripheral neuropathy, and spastic paraplegia [30]. The phenotypes associated with *MTRFR* mutations are referred to as combined oxidative phosphorylation deficiency‐7 (COXPD7) or autosomal recessive spastic paraplegia type 55 (SPG55); the former was used as the designation for this disease. COXPD7 typically manifests between the ages of 3 and 12 months and, in most cases, results in death between childhood or adolescence, with a few exceptions (51 or 53 years) [25, 27, 28].

A combination of biochemical reconstitution and cryo‐EM determined the role of MTRFR in ribosome rescue and elucidated its mechanism of action [33]. Translating mitoribosomes are split into two mitoribosomal subunits facilitated by specific ribosome‐splitting factors, although the precise cause of the split remains unclear. A section of the C‐terminal extension of MTRFR binds to the cofactor MTRES1 (C6orf203), and the MTRFR·MTRES1 complex enters the A‐site of the mtLSU to hydrolyze the peptidyl‐tRNA at the P‐site. At the same time, MTRES1 interacts with the anticodon loop of the peptidyl‐tRNA. These findings suggest MTRFR as a mitoribosome rescue factor for the abnormal split mtLSU occupied by peptidyl‐tRNA. As such, C12orf65 was named mitochondrial translation release factor in rescue, mtRF‐R or MTRFR [33]. However, a recent study focusing on the intrinsic generation of nonstop mRNA in mitochondria demonstrated that MTRFR is essential for the rescue of nonstop ribosomes but not of split mtLSU [3]. Additionally, no compensatory effects of ICT1 were observed in human *MTRFR*‐deficient fibroblasts. The rescue targets of MTRFR have not yet been fully established, making the disparity between MTRFR and ICT1 in the ribosome rescue process less clear.

Creating vertebrate models with *MTRFR* dysfunction will help to facilitate research and drug development for rare genetic diseases, such as COXPD7. To the best of our knowledge, vertebrate models with COXPD7 are lacking. In the present study, we generated zebrafish (*Danio rerio*) knockout lines of *ict1* and *mtrfr* using the CRISPR/Cas9 genome editing system, although we could not produce *Mtrfr* knockout mice (*Mus musculus*). Unexpectedly, both zebrafish knockout lines appeared healthy and fertile. We examined the effects of the knockout of each gene on mitochondrial morphology, lethality, and behavior under various stress conditions to characterize the two knockout lines. Additionally, we performed a structure‐based sequence comparison analysis of Ict1 proteins from various metazoans, revealing that teleost Ict1 proteins lack the mtLSU‐binding motif. These findings suggest that zebrafish represent a very limited category of vertebrates for which *ict1* and *mtrfr* knockout lines can be generated. We believe that this knockout zebrafish line has the potential to be used as a mode for COXPD7, if stress conditions are controlled.

## Materials and methods

### Animal care

All mice experiments were approved by the Animal Care and Experimentation Committee of Gunma University (Permit Numbers: 16‐061) and conducted in accordance with the approved guidelines. All zebrafish experiments were approved by the Animal Care and Experimentation Committee of Gunma University (23‐019, 23‐023) and the Animal Experiments Committee and Gene Modification Experiments Safety Committee of Osaka University (04294 and FBS‐14‐002‐1). Zebrafish were maintained under standard conditions of 28 °C and a 14/10‐h light/dark cycle. When necessary, mice were euthanized by cervical dislocation performed by personnel with demonstrated technical proficiency, and zebrafish were euthanized by immersion in an overdose of tricaine methane sulfonate.

### Generation of *MTRFR* knockout mice using the CRISPR/Cas9 system

C57BL/6J and ICR mice were purchased from Charles River Japan. Female C57BL/6J mice aged 5 weeks were superovulated by administering 7.5 units of pregnant mare's serum gonadotropin (PMSG; ASKA Animal Health, Tokyo, Japan), followed by 7.5 units of human chorionic gonadotrophin (hCG; ASKA Animal Health) 48 h later. Females were mated overnight with C57BL/6J male mice. The following day, fertilized eggs were collected from the oviducts. Genome editing was performed as previously described [34]. The target sequence of crRNA (5′‐GCCTCCCTCTACCACTGATC‐3′) was obtained using the web services of CRISPRdirect [35]. Equal volumes of crRNA (100 μm; IDT, Coralville, IA, USA) and *trans*‐activating crRNA (tracrRNA) (100 μm; IDT) were combined in a duplex buffer (IDT), heated in a thermal cycler to 95 °C for 5 min, and incubated for 10 min at room temperature according to the manufacturer's protocol. Combined crRNA/tracrRNA (50 ng·μL^−1^) and recombinant Cas9 protein (40 ng·μL^−1^; GeneArt Platinum Cas9 Nuclease, Thermo Fisher Scientific, Waltham, MA, USA) were injected into the cytoplasm of fertilized eggs. The injected embryos were cultured in M16 medium at 37 °C under 5% CO_2_ in air. The following day, 74 embryos developed to the 2‐cell stage were transferred into the ampulla of the oviduct of pseudopregnant ICR females. The genotypes of 19 pups were confirmed using polymerase chain reaction (PCR), followed by TA cloning and sequencing. The primer sequences are listed in Table S1.

### Generation of *ict1* and *mtrfr* knockout zebrafish lines using the CRISPR/Cas9 system

*ict1* and *mtrfr* zebrafish knockout lines were generated using the CRISPR/Cas9 genome editing system [36]. The target sequences (*ict1*: 5′‐GGAATAACGGCGCTCAGCAG‐3′, *mtrfr*: 5′‐GTTCGCGGATCTGGACCTGG‐3′) were obtained using the web services of ZiFit [37] and CRISPRdirect [35]. Template DNA fragments were prepared using PCR, and sgRNAs were synthesized and purified using *in vitro* Transcription T7 Kit (Takara, Kusatsu, Japan) following the manufacturer's protocol. A total of 1–2 nL of injection solution [100 ng·μL^−1^ sgRNA, 250 ng·μL^−1^ Cas9 protein (New England Biolabs, Ipswich, MA, USA), and 0.01% fast green dye] was injected into zebrafish fertilized eggs at the one‐cell stage. The injected F_0_ generation chimeric fish were crossed with the wild‐type fish to generate the F_1_ generation fish pool. F_1_ fish with mutation in the target site were crossed with the wild‐type to generate the F_2_ generation fish pool. The target site sequences of F_2_ generation fish were determined, and, as a result, one heterozygous loss‐of‐function mutant line of *ict1* and that of *mtrfr* were selected. Each heterozygous mutant line was crossed with each other to obtain the homozygous mutant lines, *ict1* ^−/−^ and *mtrfr* ^−/−^. The genotypes were confirmed using PCR, followed by TA cloning and sequencing.

### *In vitro* culture of cells from caudal fins, mitochondrial staining, and mitochondrial morphology analysis

*In vitro* culture of cells from caudal fins was performed as previously described [38]. Cells were isolated from the caudal fins of anesthetized adult fish and were clipped into approximately 2 mm cubes manually using a surgical knife under a stereomicroscope. The fin cells were washed five times with phosphate‐buffered saline (PBS) [137 mm NaCl (Nacalai, Kyoto, Japan), 27 mm KCl (Wako, Osaka, Japan), 100 mm Na_2_HPO_4_·H_2_O (Wako)] and dissolved in 1 mL of a 100‐fold dilution of the trypsin solution (Wako) in PBS in a 1.5 mL protein low‐binding tube (the trypsin solution: 0.5 w/v% trypsin and 5.3 mmol/L EDTA·4Na without phenol red). After incubating for 15 min at 28 °C, the sample was rinsed five times with PBS to remove trypsin and incubated with the collagenase solution for 3 h at 28 °C with gentle shaking, consisting of 1 mg·mL^−1^ collagenase I (Thermo Fisher Scientific), 0.1 mg·mL^−1^ DNase I (Worthington Biochemical, Lakewood, NJ, USA), and 1.2 mg·mL^−1^ BSA (Wako) in PBS.

Following incubation, the sample was centrifuged and washed thrice with 1 mL of PBS, and 800 μL of the supernatant was removed. A total of 120 μL of the sample was seeded into a 6‐channel slide with a nonadherent surface for fluorescence microscope (μ‐slide VI 0.4; ibidi, Gräfelfing, Bavaria, Germany) and incubated for 3 h at 28 °C. After cell adhesion, each channel was washed twice with 200 μL of PBS, and the sample was cultured for 16 h in L‐15 medium (Gibco, Waltham, MA, USA) without fetal bovine serum. Following cultivation, the sample was washed twice with PBS and stained with MitoTracker Red CMXRos working solution (Thermo Fisher Scientific) for 20 min at 28 °C according to the manufacturer's manual. After staining, the sample was gently washed thrice with PBS and fixed with 4% paraformaldehyde (Wako) in PBS. After gently washing twice with PBS, fluorescence images of the samples were captured using a fluorescence microscope (BZ‐9000; Keyence, Osaka, Japan). The images were subjected to haze reduction using software with the microscope.

The images were analyzed using Mitochondrial Network Analysis (mina) software [39], a plug‐in to imagej/fiji [40], to obtain a summary of data parameters that classify the mitochondrial morphology. The results were displayed as violin plots created using graphpad prism 9.3.1 for Windows (GraphPad Software, Boston, MA, USA).

### Behavioral assay

For motility assay, 7 dpf larvae of the wild‐type, *ict1* ^−/−^, and *mtrfr* ^−/−^ were used. Ten larvae from each line were placed in a sterile petri dish (Diameter 90 × Height 20 mm; Sansei Medical, Kyoto, Japan), and their movements were photographed for approximately 30 s using a 60 fps digital camera with 12.1 megapixels resolution (Canon PowerShot S120; Canon, Tokyo, Japan). All movement recordings began 10 min following adaptation in visible light in an air‐conditioned room (25 °C).

The video in MPEG3 format was analyzed using homemade tracking software developed in python 3.0 using the OpenCV library (Python Software Foundation, Wilmington, DE, USA). A JPG image was extracted from each video frame, and in each image, the positional information of a selected larva was derived from the contrast of the larva's head against the background. The data from each image were subsequently integrated to obtain data on the temporal variations in the position of the larva.

The mean swimming speed of each larva and the ratio of stop time to total measurement time were calculated. Item definitions and calculation methods are discussed in the Results section.

A touch response assay was conducted on the 7 dpf larvae in a petri dish. The larval movements triggered by touching the dorsal surface of the tail with an eyelash probe were monitored. The remaining operations were the same as the motility assay, except the recording time was 1 min. Responses immediately after touching were classified into three types based on distinct movement patterns (see the Results section). The proportion of each type in each line is shown as a 100% stacked bar chart.

## Results

### Generation of *Mtrfr* knockout mouse line and its lethality

Gene and protein designations of the mitoribosome rescue factors for each species used in this study are listed in Table S2. For representative names, we employed the designations in humans, such as *ICT1* and ICT1; *MTRFR* and MTRFR.

We first attempted to generate *Mtrfr* knockout mouse line on a wild‐type background using the CRISPR/Cas9 genome editing system and obtained three independent lines of *Mtrfr* heterozygous knockout mouse (*Mtrfr* ^+/−^) (Table 1, Fig. S1). In each knockout line, a deletion mutation was introduced into exon 3, resulting in a frameshift and the generation of a premature termination codon (PTC). The three mutant alleles encoded truncated proteins (residues 54, 53, and 57) instead of the full‐length 84‐residue protein, all of which lacked the GGQ domain and C‐terminal extension (Fig. S1). All the heterozygous knockout lines appeared normal, consistent with the fact that in humans, the mitochondrial disease, COXPD7, resulting from loss‐of‐function mutations in *MTRFR* is inherited in an autosomal recessive manner. However, no homozygous knockout mouse lines (*Mtrfr* ^−/−^) could be generated from each cross of the three heterozygous knockout lines, indicating that the *Mtrfr* knockout was lethal in mice (Table 1). In the heterozygous mouse crosses, the number of offspring at birth remained unchanged without subsequent death, implying that the homozygous knockout lines were not originally born and thus embryo‐lethal. This finding in mice contrasts with the finding that loss‐of‐function mutations in MTRFR are not lethal and cause human disease. In gene knockout experiments, the phenotype of the knockout mutant is often different or not completely identical in mice and humans.

**Table 1:** Results of each cross of the three lines of heterozygous *Mtrfr* knockout mice. The mutated genome sequences of the three knockout lines are shown in Fig. S1.

*Mtrfr*	+/+	+/−	−/−
#1	22	32	0
#2	42	30	0
#3	20	34	0

### Generation of *ict1* and *mtrfr* knockout zebrafish lines and their nonlethality

**Fig. 1:**
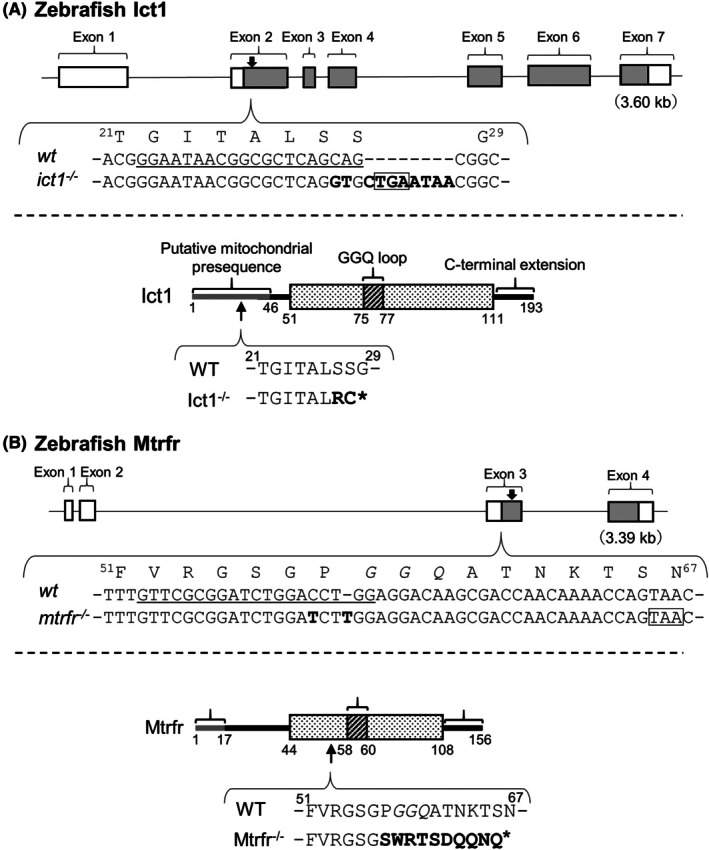
Generation of *ict1* and *mtrfr* knockout zebrafish lines using the CRISPR/Cas9 system. (A) Upper panel: Schematic diagram of the configuration of *ict1* from zebrafish according to the Ensemble database [56]. Exons are represented as boxes, with gray regions indicating the protein‐coding regions. The downward arrow indicates the mutation position. Only the DNA sequences of genes for the wild‐type (*wt*) and *ict1* ^−/−^ before and after the mutation position are shown, and the corresponding amino acid sequence for *wt* is shown above the DNA sequence. The guide RNA sequence in *wt* is underlined. The mutated sequence in *ict1* ^−/−^ is presented in bold, and the boxed sequence indicates a premature stop codon. Bottom panel: A schematic domain representation of the Ict1 protein. The structured catalytic domain (the GGQ domain) is represented as a box based on the mammal ICT1 structures. The gray bar indicates the region corresponding to a putative mitochondrial targeting sequence or presequence, which was predicted using the TargetP‐2.0 web server [57]. The upward arrow indicates the first amino acid that undergoes alteration due to the mutation. The amino acid sequences for the *wt* and *ict1* ^−/−^are shown. Bold letters indicate mutated residues in *ict1* ^−/−^, and an asterisk indicates termination. (B) The figure regarding *mtrfr* is shown. The notation is the same as (A).

We next generated *ict1* and *mtrfr* knockout zebrafish lines on a wild‐type (Tu) background using the CRISPR/Cas9 system. To this end, we targeted exons 2 and 3 in *ict1* and *mtrfr*, respectively, to disrupt the translation of the full‐length proteins (Fig. 1). First, a heterozygous mutant line of *ict1* and that of *mtrfr* was established, and the homozygous mutant lines were obtained following next cross‐experiments.

We obtained one homozygous mutant line of *ict1* and one of *mtrfr*. For *ict1* knockout, three bases in exon 2 were replaced with 11 bases (Fig. 1A), while for *mtrfr* knockout, one‐base change and one‐base insertion were made in exon 3 (Fig. 1B). These mutations cause frameshifts and introduce a PTC into *ict1* and *mtrfr*.

If the mutant genes of *ict1* and *mtrfr* are expressed, the resulting expressed proteins will consist of 28 and 66 amino acid residues as opposed to the normal full‐length proteins of 193 and 156 residues, respectively (Fig. 1A,B). Both proteins are devoid of the GGQ domain and the C‐terminal extension that follows. These findings indicate that one knockout line for *ict1* and *mtrfr* was created, termed *ict1* ^−/−^ nor *mtrfr* ^−/−^, respectively.

Neither adult *ict1* ^−/−^ nor *mtrfr* ^−/−^ lines exhibited any apparent differences from the wild‐type in morphology, size, or behavior for at least 6 months following fertilization under standard breeding conditions (Fig. S2). Additionally, the *ict1* ^−/−^ and *mtrfr* ^−/−^ lines had normal reproductive capacity. These results indicate that the growth and development of zebrafish under normal or nonstress conditions do not require both genes.

### Mitochondrial morphology of adult caudal fin cells differs between the wild‐type *ict1* ^−/−^ and *mtrfr* ^−/−^

**Fig. 2:**
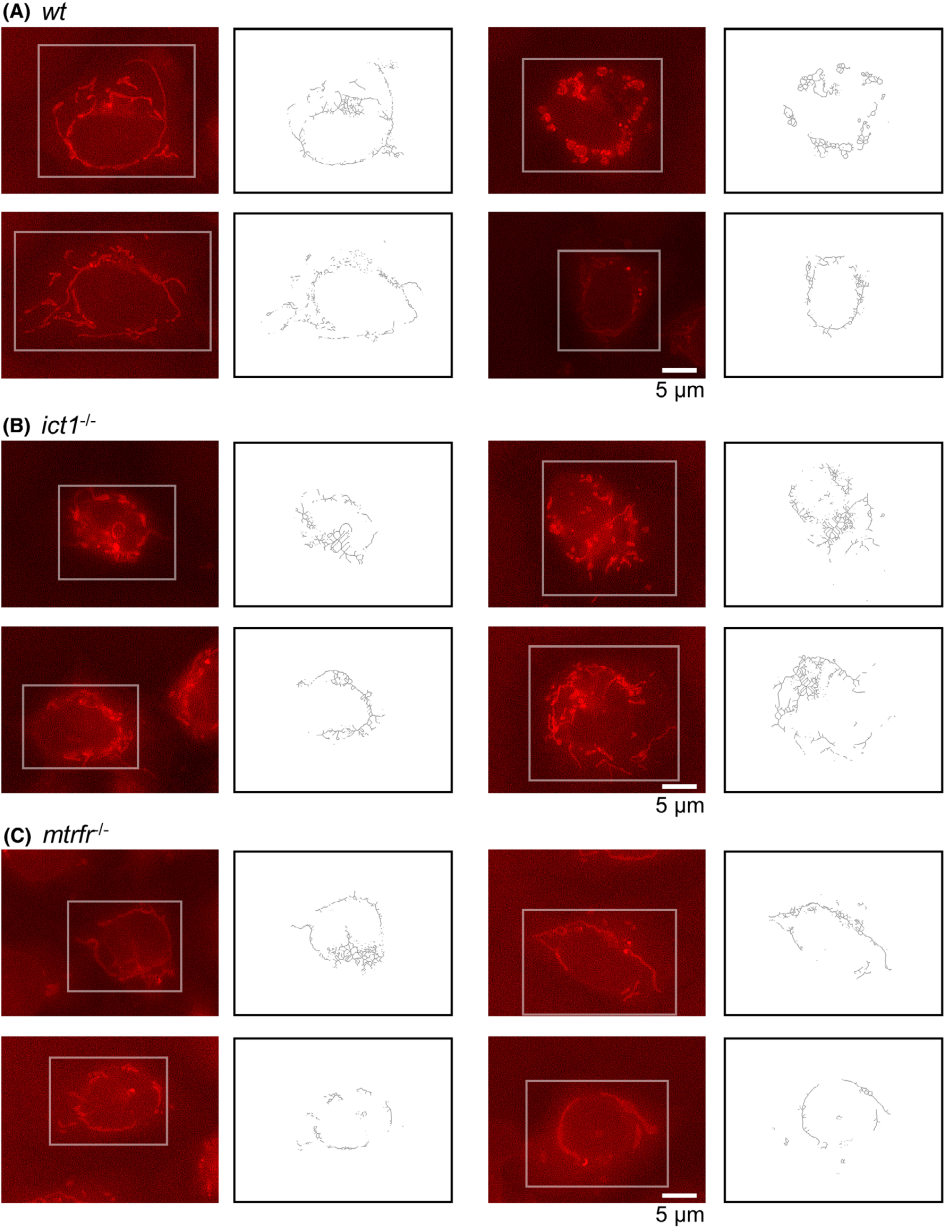
Mitochondrial morphology in the caudal fin cells from adult fish of the wild‐type, *ict1* ^−/−^, and *mtrfr* ^−/−^. Four representative fluorescent images stained with MitoTracker Red CMXRos (left) and the corresponding skeletonized images created using the mina software (right) each for the caudal fin cells from adult fish of the wild‐type (A), *ict1* ^−/−^ (B), and *mtrfr* ^−/−^ (C). These images were selected from those used for the analysis presented in Fig. 3. Each scale bar represents 5 μm. Mitochondria in a single cell framed in white in the left panels are skeletonized using the mina software.

Notwithstanding the absence of any observable abnormalities in the knockout individuals, the deletion of *ict1* or *mtrfr* should have some influence on mitochondrial function in zebrafish cells. We examined mitochondrial morphology in keratinocyte‐like cells from the adult caudal fins of *ict1* ^−/−^ and *mtrfr* ^−/−^ stained with MitoTracker Red and captured images using a fluorescence microscope (Fig. 2).

Mitochondrial morphology in the fluorescence images was analyzed using mina software [39], an imagej/fiji plugin [40]. Briefly, mina first binarizes the original image and skeletonizes it to generate a morphological skeleton (Fig. 2). This process allows for the extraction of quantitative parameters that precisely capture mitochondrial morphology. mina employs a system that classifies objects in a skeleton image into two categories. Rods and punctate shapes are classified as ‘individuals’, while all objects with at least one junction as ‘networks’ (Fig. S3). Finally, mina produces a series of outputs of mitochondrial descriptors: number of individuals and networks, mean branches per network (network size), and rod/branch lengths; mitochondrial footprint (skeletal area calculated prior to skeletonization).

**Fig. 3:**
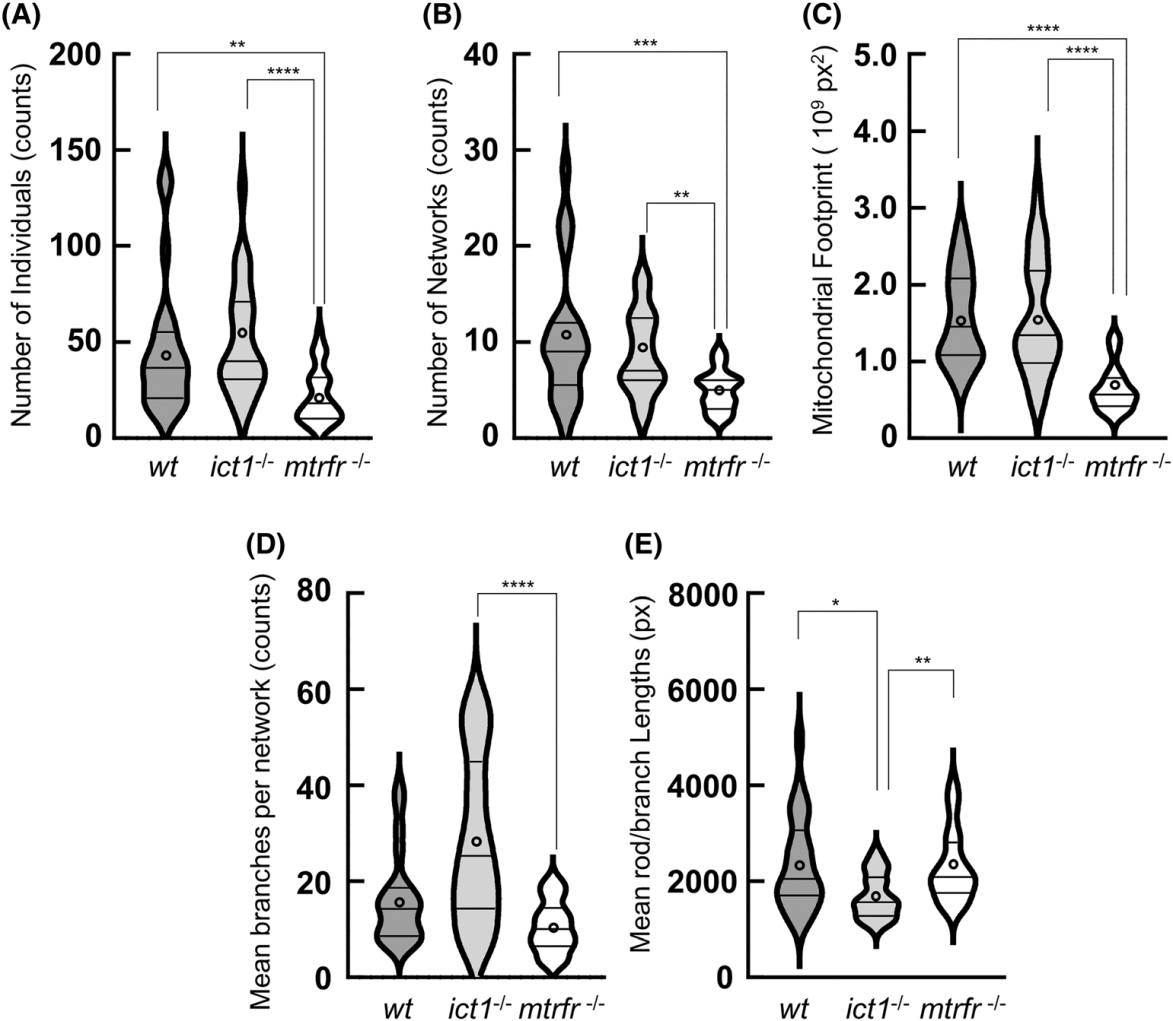
Mitochondrial network analysis for the caudal fin cells from adult fish of the wild‐type, *ict1* ^−/−^, and *mtrfr* ^−/−^. Skeletonized images shown in Fig. 2 were analyzed using the mina software to show mina descriptors: number of individuals (A), number of networks (B), mitochondrial footprint (C), mean branches per network (D), and mean rod/branch lengths (E). Violin plots are shown for the four descriptors of *wt* (*n* = 22), *ict1* ^−/−^ (25) and *mtrfr* ^−/−^ (29). The violin plot outlines illustrate kernel probability density; in other words, the width of the violin plot represents the proportion of the data, thus demonstrating data distribution. The third quartile, the median, and the first quartile are indicated by horizontal bars from top to bottom, while the average is indicated by a circle. Asterisks indicate significant differences between individual groups (indicated by bars). Kruskal–Wallis test with Dunn's multiple comparisons test, ****P < 0.0001; ***P < 0.001; **P < 0.01; *P < 0.05.

Comparison of the mitochondrial descriptors among the wild‐type, *ict1* ^−/−^, and *mtrfr* ^−/−^ showed marked differences in mitochondrial morphology between each knockout line and the wild‐type and between the knockout lines. In *ict1* ^−/−^ cells, no significant changes were observed in the number of individuals and networks and the mitochondrial footprint compared with the wild‐type (Fig. 3). However, the rod/branch length significantly decreased, while the number of branches per network increased, with an average twice that of the wild‐type; although *P* = 0.0638. Conversely, in *mtrfr* ^−/−^ cells, no significant changes were observed in rod/branch length and the number of branches per network compared with wild‐type (Fig. 3). However, the number of individuals and networks as well as the footprint significantly decreased compared with wild‐type. Taken together, the *ict1* deletion affected the network properties to a greater extent than the number of individuals and networks, whereas the *mtrfr* deletion exhibited the opposite effect. Since *ict1* ^−/−^ and *mtrfr* ^−/−^ appeared healthy, the altered mitochondrial morphology may be linked to compensatory mechanisms for the putative mitochondrial defects caused by each gene deletion.

### Starvation reduces the survival rates of *ict1* ^−/−^ and *mtrfr* ^−/−^ larvae

Under nonstressed conditions, neither deletion of *ict1* nor *mtrfr* substantially impacted the development or growth of zebrafish. Therefore, we examined the survival rate of the *ict1* ^−/−^ and *mtrfr* ^−/−^ lines of zebrafish larvae when subjected to starvation conditions. Stress conditions and changes in energy sources have various effects on mitochondria, such as changes in the level of OXPHOS and mitochondrial morphology and biogenesis [41, 42]. Ten larvae at 3‐day postfertilization (dpf) were maintained in Petri dishes without food. The breeding conditions helped to minimize the potential for contamination of the breeding water. The number of living larvae was counted daily. The larvae are initially nourished by the yolk sac, which is fully absorbed by approximately 10 dpf [43]. Consequently, the larvae are subjected to complete starvation stress for the first time after this period.

**Fig. 4:**
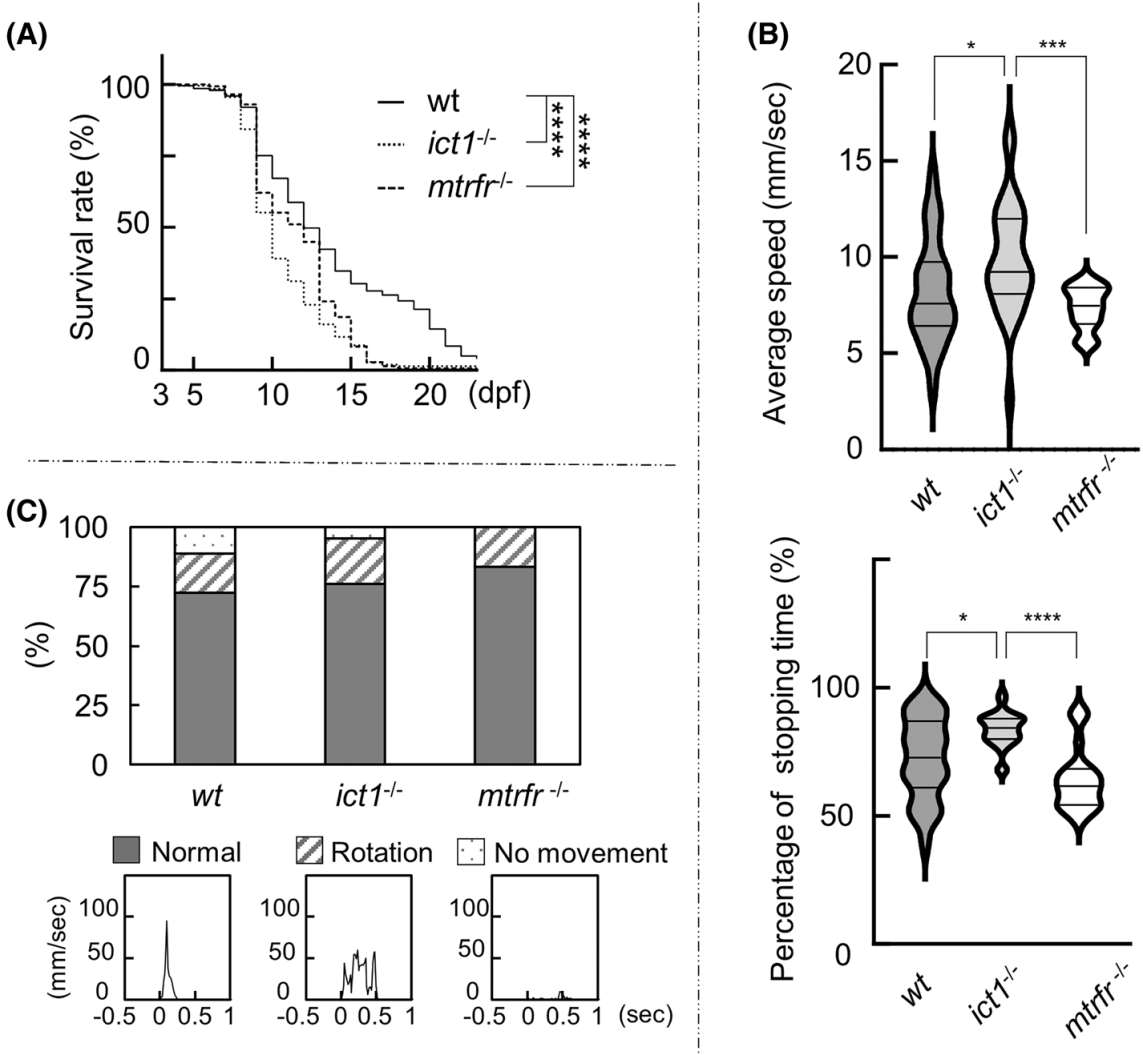
Survival rates and motor behaviors of larvae of the wild‐type, *ict1* ^−/−^, and *mtrfr* ^−/−^. (A) Kaplan–Meier survival curves of the wild‐type (*n* = 201), *ict1* ^−/−^ (205), and *mtrfr* ^−/−^ (145) reared in Petri dishes without feed. The observations were initiated on 3 dpf. Asterisks indicate significant differences between individual groups (Kruskal–Wallis test with Dunn's multiple comparisons test). ****P < 0.0001; ***P < 0.001; **P < 0.01; *P < 0.05. (B) Upper: Average speed at which the larvae of the three lines were moving, calculated by dividing the distance traveled by the time spent traveling. Violin plots show the average speed of the 7 dpf larvae of the wild‐type (*n* = 29), *ict1* ^−/−^ (30), and *mtrfr* ^−/−^ (24). Calculation of the travel time does not include frames in which the larvae ceased their travel (i.e., travel distance < 0.02 mm). The third quartile, the median, and the first quartile are indicated by horizontal bars from top to bottom. Data were assessed using the Kruskal–Wallis test with Dunn's multiple comparisons test. Movement trajectories of the larvae are shown in Fig. S4B. Bottom: Percentage of stopping time during which the larvae ceased to travel, calculated by dividing the time during which the larvae ceased their travel (i.e., travel distance < 0.02 mm) by the total recording time. (C) Upper: Percentage of three types of touch response to the 7 dpf larvae of the three lines classified into normal, rotation, and no movement. Bottom: a typical plot (seconds vs. speed) for each category is shown. A more detailed illustration of the classification is shown in Fig. S4C. No significant differences between *wt* and *ict1* ^−/−^ (Fisher's exact test, *P* = 0.764) or between *wt* and *mtrfr* ^−/−^ (*P* = 0.703) were observed.

Kaplan–Meier survival curves showed significant differences between the wild‐type and *ict1* ^−/−^ and between the wild‐type and *mtrfr* ^−/−^ (Fig. 4A). Up to 8 dpf, the survival rate was similar among the three lines, while at 10 dpf, the order of survival rate was *wt* (75%) > *ict1* ^−/−^ (55%) > *mtrfr* ^−/−^ (39%), while at 13 dpf, that is: *wt* (40%) > *ict1* ^−/−^ ≈ *mtrfr* ^−/−^ (24%). *mtrfr* ^−/−^ appeared to be more sensitive to starvation than *ict1* ^−/−^. The maximum life span of the wild‐type was 23 dpf, whereas that of the two knockout lines was 18 dpf. These results indicate that ICT1 and MTRFR play essential roles in the survival of larvae under conditions of starvation stress. In other words, under such stress conditions, the necessities of ICT1 and MTRFR may become apparent.

### Ict1^−/−^ and Mtrfr^−/−^ larvae exhibit altered locomotor behaviors, whereas touch remains unaffected

The clinical traits of COXPD7 include optic atrophy, peripheral neuropathy, and spastic paraparesis. We first performed behavioral assays for *ict1* ^−/−^ and *mtrfr* ^−/−^ larvae: locomotor activity (swimming speed and stopping time during movement) and touch response. To examine locomotor activity, we monitored the movement of 7 dpf larvae in Petri dishes for approximately 30 s using a 60 fps digital camera with 12.1 megapixels resolution (Fig. S4A,B). Seven dpf larvae of the wild‐type and knockout lines were under little or no starvation stress [43]. All movement recordings started 10 min following adaptation in visible light at room temperature. The total distance traveled by each larva was calculated using homemade tracking software. Mean swimming speed was determined by dividing the distance traveled by the elapsed time obtained using 1/60 s per flame, excluding frames in which the larvae exhibited no substantial movement (< 0.02 mm). The ratio of stopping time to measurement time was determined by dividing the number of frames in which the larvae exhibited no movement by the total number of frames. That is, as the ratio value approached 1, the proportion of time during which zebrafish exhibited no movement increased.

The median value of the mean swimming speed of *ict1* ^−/−^ was higher than that of the wild‐type (Fig. 4B, upper), with a wider distribution. The median value of the mean stopping time of *ict1* ^−/−^ was higher, with a narrower distribution (Fig. 4B, lower). In contrast, the median value of the mean swimming speed of *mtrfr* ^−/−^ was similar to that of the wild‐type, with a narrower distribution (Fig. 4B, upper). The median of the mean stopping time of *mtrfr* ^−/−^ was lower than that of the wild‐type, with a distribution that contained a major and minor peak, differing from the wild‐type distribution (Fig. 4B, bottom). Overall, *ict1* ^−/−^ showed a tendency to swim relatively faster and stop less frequently than the wild‐type, whereas *mtrfr* ^−/−^ tended to have comparable speed and increased stopping frequency compared to the wild‐type.

A touch response assay was conducted on 7 dpf larvae in a Petri dish by touching the dorsal portion of the tail with an eyelash probe. Responses immediately after touching were classified into three types based on their distinct movement patterns (Fig. S4C). Comparisons of touch responses between the three lines showed no significant differences based on this classification (Fig. 4C).

Collectively, except for the touch response, significant differences in locomotor behavior among *ict1* ^−/−^, *mtrfr* ^−/−^, and wild‐type line larvae were observed. These results suggest that either gene deletion of *ict1* or *mtrfr* has a greater effect on the functions of neuron cells than on those of muscle cells. Petri dishes may have stressed the larvae by limiting the range of movement, especially depth. Therefore, this stress might have attributed to the differences in locomotor behavior between the wild‐type and knockout lines.

### Zebrafish Ict1 lacks a mitoribosome‐binding motif found in most eukaryotic ICT1 proteins

**Fig. 5:**
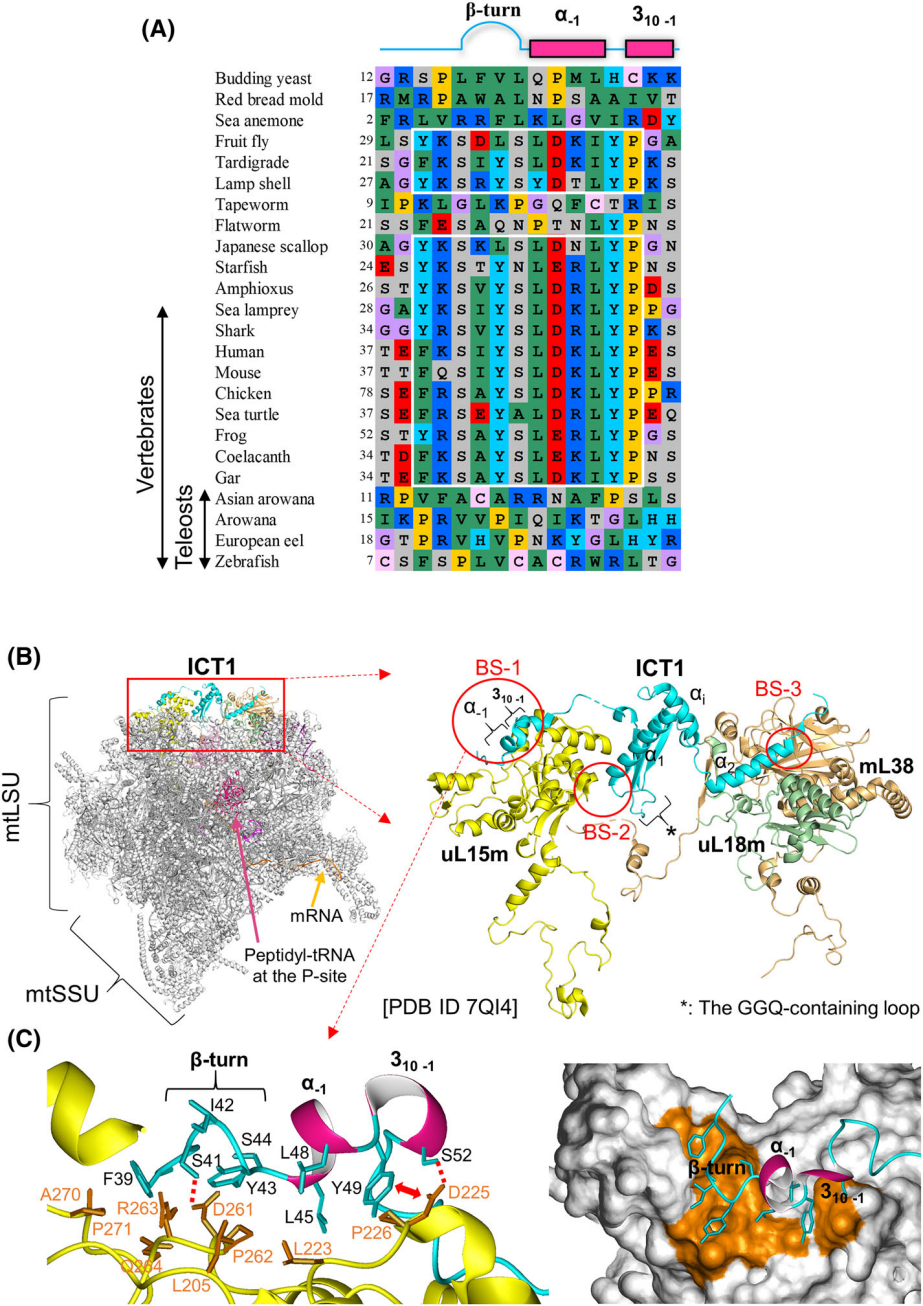
Binding of human ICT1 to a mitoribosome as an integral ribosomal protein and the characteristic binding motif at the N terminus. (A) Amino acid sequence alignment resides for the YSLDK motif at the N terminus among ICT1 proteins from various eukaryotes. The YSLDK motif resides are indicated by a box with white lines. Above the alignment, the secondary structure elements formed by the YSLDK motif residues found in human ICT1 are shown. For *C. elegans* ICT1, the N‐terminal region is short; therefore, the sequence is not shown. The alignments were performed using the clustalw program [58]. Alignment of the full‐length sequence of the ICT1 proteins is shown in Fig. S5. Accession codes for the ICT1 proteins used are provided in the legend of Fig. S5. The numbers to the left of the alignment correspond to the number of the first amino acid in each ICT1 protein. Alignments are colored as follows: purple: glycine (G); yellow: proline (P); green: small and hydrophobic amino acids (A, V, L, I, M); pink: hydrophobic aromatic residues (F, W); gray: hydroxyl and amine amino acids (S, T, N, Q); red: negatively charged amino acids (D, E); blue: positively charged amino acids (K, R); pale pink: cysteine (C); cyan: histidine (H) and tyrosine (Y). (B) Left: Overall structure of a human mitoribosome containing mRNA, tRNA in the P‐site, and ICT1 as a ribosomal protein in a ribbon representation. The structures in this figure are all based on PDB ID 7QI4 [44]. Ribosomal proteins and rRNAs in the mitoribosome are depicted in gray, while the others are indicated by arrows. Right: costructure of ICT1 (aquamarine) and the three ribosomal proteins, uL15m (yellow), uL18m (forest), and mL38 (light brown), which are extracted and engaged from the structure in the left panel. BS‐1, ‐2, and ‐3 (circled in red) indicate the major binding sites of ICT1 to the three ribosomal proteins. The structure of BS‐1 is illustrated in (C), while those of BS‐2 and BS‐3 are in Fig. S6. The program pymol (PyMOL Molecular Graphics System, Version 2.0, Schrödinger, LLC) was used to visualize and analyze the structures. (C) Left: Enlarged view of BS‐1. Side chains of nine residues of the characteristic binding motif of ICT1, the YSLDK motif, are shown; α‐ and 3_10_‐helixes are depicted in pink. Side chains of residues in uL15m interacting with the motif residues are shown. Red dotted lines show a putative hydrogen bond, while a two‐red way arrow indicates a putative anion‐π interaction. Right: Molecular surface representation showing the interaction region of uL15m (depicted by orange) with a ribbon representation of the YSLDK motif residues. The program molmol [59] was used to visualize and analyze the structures.

As *ICT1* deletion induced a marked difference in lethality between zebrafish and mammals, we speculate that fundamental differences exist in function and, thus, amino acid sequence between the zebrafish and mammalian ICT1 proteins. As such, we performed a sequence alignment among ICT1 proteins from various metazoans (Fig. 5A, Fig. S5). In the light of the structure of the human mitoribosome [44] (Fig. 5B), an mtLSU‐binding sequence was identified at the N terminus of most ICT1 proteins; however, this motif is not found in teleosts, including zebrafish (Fig. 5A). The consensus sequence for mtLSU binding is as follows: F/Y‐K‐S‐X‐Y‐S‐L‐D‐K/R‐L‐Y‐P (where X represents hydrophobic residues in many instances), henceforth called the YSLDK motif for convenience. In the mitoribosome structure, the motif residues form a single stretch of structure consisting of three consecutive minimal structural units: a β‐turn (type I, one of the most common β‐turns), a one‐turn of an α‐helix, and a 3_10_‐helix [44] (Fig. 5C, left). This motif structure facilitates the assembly of the hydrophobic side chains of the residues in one direction. The resulting hydrophobic surface in ICT1 interacts with the surface formed by specific hydrophobic residues of mitochondrial ribosomal protein uL15m (MRPL15) (Fig. 5C, right). Additionally, two putative hydrogen bonds are formed between Ser41 in ICT1 and Asp261 in uL15m and between Ser52 and Asp225, with a possible anion‐π interaction between Tyr49 and Asp225 (Fig. 5C, left). A series of interactions indicates a highly specific interaction between the YSLDK motif and uL15m structures; they appear to be well conserved in most metazoans (i.e., other metazoans except for teleosts).

In addition to the binding site of the motif to mtLSU (termed BS‐1), two other binding sites, BS‐2 and BS‐3, governed by hydrophobic interactions were identified [44] (Fig. 5B, Fig. S6). Generally, hydrophobic interactions are pivotal in the ‘permanent interaction’ between proteins; in this case, between ICT1 and certain ribosomal proteins [45]. Specifically, in BS2, Tyr142 and Phe144 in ICT1 interact with Ala145, Tyr240, and Leu162 in uL15m. In BS‐3, Leu188 in ICT1 interacts with Leu137 in uL18m (MRPL18) and Pro186 and Tyr188 in mL38 (MRPL38) (Fig. S6). The hydrophobic residues that constitute the two sites, as seen in BS1, are highly conserved in ICT1 proteins and the ribosomal proteins from most metazoans (Figs S5 and S7).

However, ICT1 proteins from teleosts lack the YSLDK motif, and the N‐terminal sequence upstream of the GGQ domain is not preserved among teleosts (Fig. 5A, Fig. S5). Regarding BS‐2 and BS‐3, some residues differ between most metazoans and teleosts (e.g., Leu162 in uL15m vs. Gly/Lys/Ala; Phe144 in ICT1 vs. Gln/Arg/His) (Figs S6 and S7), and therefore, hydrophobic interactions as in the human mitoribosome may not be formed in the teleost counterparts. These results combined suggested that ICT1 in teleosts cannot bind to mtLSU as a component of the mitoribosome. However, the possibility that zebrafish ICT1 interacts with mtLSU in different modes or different mtLSU regions cannot be excluded.

## Discussion

In this study, we obtained three independent lines of heterozygous *Mtrfr* knockout mice but could not obtain homogenic knockout mice. Even if the *MTRFR* mutant genes are expressed, the three mutation positions enable the mutant proteins to lack amino acid residues that constitute the GGQ domain and the C‐terminal extension. Similar mutation positions have been reported in patients with COXPD7, such as Pro34Ilefs*25 [28] and Thr3Argfs*54 [23]. This indicates that the negative effects of gene deletions that effectively negate MTRFR expression are likely more pronounced in mice than in humans. Since *Ict1* deletion is lethal in mice, mitoribosome rescue factors, ICT1 and MTRFR, are essential for normal development and growth in mammals.

We generated one knockout zebrafish line each for *ict1* and *mtrfr*, which appeared healthy, contrary to expectations. However, under starvation conditions, the survival rates of larval knockout lines were significantly lower than wild‐type. The production of ATP in mitochondria requires the translation of all genes encoded by mtDNA, whose proteins are subunits of five multimeric complexes of the OXPHOS chain. For larvae to survive in nutrient‐poor environments, mitochondrial translation must proceed efficiently and without interruption, exceeding the efficiency seen in nonstress conditions. These conditions highlight the necessity for ribosome rescue factors.

Next, we address the following question: why were the two zebrafish knockout lines viable and healthy under nonstressed conditions? One possible answer is that different mechanisms that compensate for the loss of either factor become activated in knockout cell lines. The mina analysis of mitochondria in adult caudal fin cells showed significant differences in mitochondrial morphology between each of *ict1* ^−/−^ and *mtrfr* ^−/−^ and the wild‐type and between *ict1* ^−/−^ and *mtrfr* ^−/−^. Since the two knockout lines are healthy, these differences in mitochondrial morphology from the wild‐type may be beneficial for *ict1* ^−/−^ and *mtrfr* ^−/−^ cells. The most notable feature of mitochondrial morphology observed in *ict1* ^−/−^ was the increase in the network size without an increase in the number of networks. This suggests activated mitochondrial fusion or a more fused mitochondrial network, based on the quantification results of mitochondrial morphology in different patient‐derived fibroblasts, including those affected by mitochondrial diseases, analyzed using mina [39, 46, 47]. In contrast, the most notable feature of mitochondrial morphology in *mtrfr* ^*−/−*^ cells was the significant reduction in the number of individuals and networks and mitochondrial footprint. Such reductions often indicate mitochondrial fission or fragmentation, which is linked to mitochondrial dysfunction [48, 49, 50]. However, at the same time, mean rod/branch lengths in *mtrfr* ^*−/−*^ cells increased to some extent than that of the wild‐type, and this may partially help compensate for the putative mitochondrial dysfunction in *mtrfr* ^*−/−*^ cells. Further experiments are required to elucidate the compensatory mechanism; once understood, the functional differences between the two proteins will become clear.

Another potential explanation is that zebrafish Ict1 and Mtrfr share some functional similarities, enabling one factor to partially compensate for the dysfunction or loss of the other, though not entirely. Such a functional complementarity is not observed in the human counterparts, since *ICT1* deletion is lethal, while that of *MTRFR* causes mitochondrial diseases. One major reason for the lack of functional complementarity between the two rescue factors in humans is that ICT1 is a component of mtLSU (Fig. 5B), which precludes the possibility of MTRFR being a substitute for ICT1. The findings of the sequence alignments suggest that, in zebrafish, Ict1 is not a component of mtLSU; hence, the loss of Ict1 may be partially compensated for by Mtrfr.

The lethality of *ICT1* or *MTRFR* knockout line from humans, mice, zebrafish, and budding yeast is summarized in Table 2. Notably, the findings in zebrafish were similar to those in *Saccharomyces cerevisiae*. An *S. cerevisiae* mutant strain lacking either the ICT1 or MTRFR ortholog (Pth4 or Pth3, respectively) is viable under nonstress conditions. In contrast, under temperature and antibiotic stress conditions, either Pth4 or Pth3 is indispensable for growth, indicating a genetic interaction between *pth4* and *pth3* that involves functional complementarity [51]. Moreover, the ICT1 ortholog lacks the YSLDK motif and is not observed in the cryo‐EM mitoribosome structure of yeast [52] or *Neurospora crassa* [53]. This suggested that ICT1 orthologs are not components of the mitoribosomes across fungi. Thus, there may be a link between lethality or disease due to the deletion of each ribosome rescue factor, the ribosome‐binding properties of ICT1, and the presence of the YSLDK motif in specific biological groups, such as fungi and teleosts.

**Table 2:** Summary of lethality of knockout of the *ICT1* or *MTRFR* gene under normal conditions and of the presence of the YSLDK motif in different eukaryotes.

Species	*ICT1* ^−/−^	*MTRFR* ^−/−^	YSLDK motif
*Homo sapiens*	Lethal^a^	Disease	✓
*Mus musculus*	Lethal	Lethal^b^	✓
*Danio rerio*	Non‐lethal^b^	Non‐lethal^b^	–
*Saccharomyces cerevisiae*	Non‐lethal	Non‐lethal	–

^a^ Expectations from background data described in the Introduction.

^b^ Data shown in this study.

This study also indicated that, although all eukaryotes possess ICT1, teleosts (infraclass Teleostei) are the only group of vertebrates in which ICT1 lacks the YSLDK motif (Fig. S8). This uniqueness reflects the occurrence of the third genome duplication, which took place solely in teleosts, known as the teleost‐specific genome duplication (TGD) [54, 55]. Following TGD occurrence in the teleost ancestor, the YSLDK motif sequence of ICT1 appears to have undergone extensive mutations, preventing ICT1 from being a structural component of mtLSU. In contrast, the GGQ motif is fully conserved in ICT1 proteins across all eukaryotic species; notably, the GGQ motif residues do not serve as an interaction site with the mitoribosome in humans [44]. This conservation highlights the significance of ICT1 as a mitoribosome rescue factor in eukaryotes. Our findings suggest that ICT1 functions as a rescue factor under specific conditions, even in species such as humans, where it also serves as a structural component of mtLSU.

## Conflict of interest

The authors declare no conflict of interest.

## Peer review

The peer review history for this article is available at https://www.webofscience.com/api/gateway/wos/peer‐review/10.1002/2211‐5463.70054.

## Author contributions

NN and MW designed the study. MW generated the zebrafish knockout lines. CT, SH, HS, and NN performed experiments with the zebrafish lines. TH and IH generated the heterozygous mouse knockout lines. MA, SA, NN, and YI performed cross‐experiments of each heterozygous knockout line. NA created the software. KK and NN performed structural analysis. NN wrote the manuscript. KK, NA, YI, TH, IH, and MW revised the manuscript.

## Supporting information


**Fig. S1.** Generation of *Mtrfr* knockout lines of mice.
**Fig. S2.** Representative brightfield images of adult male and female zebrafish of the wild‐type, *ict1* ^−/−^, and *mtrfr* ^−/−^ lines.
**Fig. S3.** Example of mitochondrial features recognized using the mina software.
**Fig. S4.** Behavioral assays for the larvae of the wild type, *ict1* ^−/−^, and *mtrfr* ^−/−^.
**Fig. S5.** Sequence alignment of ICT1 proteins among a diverse range of eukaryotes.
**Fig. S6.** Hydrophobic interactions between ICT1 and ribosomal proteins regarding BS‐2 and BS‐3.
**Fig. S7.** Sequence alignment of uL15m, uL18m, and mL38 among metazoans.
**Fig. S8.** Simplified phylogeny tree of metazoans indicating the presence of the YSLDK motif.
**Table S1.** Primers used for PCR experiments to confirm the genotypes of mice or zebrafish in this study.
**Table S2.** Summary of the symbols of the genes and proteins for the two ribosome rescue factors in different eukaryotes.

## Data Availability

The data that support the findings of this study are available from the corresponding author upon reasonable request.
