# Supplementary material for: Knockout of the mitoribosome rescue factors Ict1 or Mtrfr is viable in zebrafish but not mice: compensatory mechanisms underlying each factor's loss
Source: FEBS Open Bio. 2025 May 16;15(8):1303–18. doi: 10.1002/2211-5463.70054 (PMC12319706; doi:10.1002/2211-5463.70054)
Supplement: Supplementary file 1 — Fig. S1. Generation of Mtrfr knockout lines of mice. Fig. S2. Representative brightfield images of adult male and female zebrafish of the wild‐type, ict1 −/−, and mtrfr −/− lines. Fig. S3. Example of mitochondrial features recognized using the mina software. Fig. S4. Behavioral assays for the larvae of the wild type, ict1 −/−, and mtrfr −/−. Fig. S5. Sequence alignment of ICT1 proteins among a diverse range of eukaryotes. Fig. S6. Hydrophobic interactions between ICT1 and ribosomal proteins regarding BS‐2 and BS‐3. Fig. S7. Sequence alignment of uL15m, uL18m, and mL38 among metazoans. Fig. S8. Simplified phylogeny tree of metazoans indicating the presence of the YSLDK motif. Table S1. Primers used for PCR experiments to confirm the genotypes of mice or zebrafish in this study. Table S2. Summary of the symbols of the genes and proteins for the two ribosome rescue factors in different eukaryotes. [file FEB4-15-1303-s001.pdf]

Supporting information

Knockout of the mitoribosome rescue factors Ict1 or Mtrfr is viable in zebrafish but not mice: compensatory mechanisms underlying each factor's loss

Nobukazu Nameki, Chika Tomisawa, Soichiro Hoshino, Hidehiko Shimizu, Masashi Abe, Sho Arai, Kanako Kuwasako, Naoki Asakawa, Yusuke Inoue, Takuro Horii, Izuho Hatada, and Masakatsu Watanabe

This file contains Figures S1-S8 and Tables S1-S2.

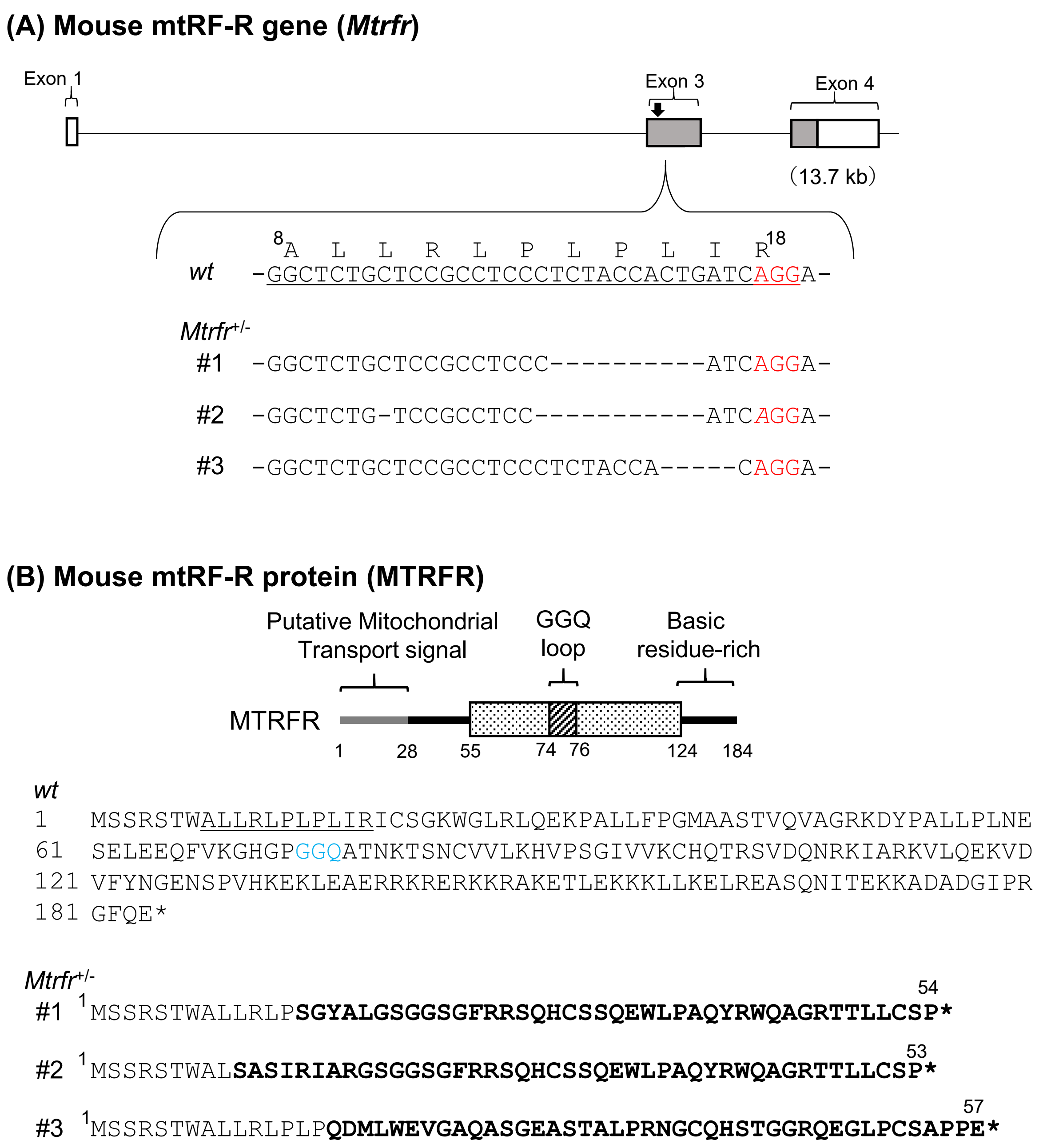

Fig. S1. Generation of *Mtrfr* knockout lines of mice.

(A) Schematic diagram of the configuration of *Mtrfr* from mouse according to the Ensemble database [56]. Exons are represented as boxes, in which gray regions indicate the protein coding regions. A downward arrow indicates the mutation position in heterozygous knockout mice (*Mtrfr*$^{+/-}$). Part of the sequences of *Mtrfr* for the wild-type (*wt*) and three lines of *Mtrfr*$^{+/-}$ are shown; the guide RNA sequence in *wt* is underlined, and the PAM sequence is shown in red. The corresponding amino acid sequence for *wt* is shown above the DNA sequence. Deleted sequence in *Mtrfr*$^{-/-}$ is presented in hyphen. (B) A schematic domain representation of MTRFR protein from mouse. The structured catalytic domain (the GGQ domain) is represented as a box. The gray bar indicates the region corresponding to a putative mitochondrial targeting sequence or pre-sequence, which was predicted using the TargetP-2.0 web server [57]. The full-length amino acid sequence is shown for the wild-type, while, if expressed, the putative sequences are shown for the three lines of *Mtrfr*$^{+/-}$. Bold letters indicate mutated residues in *Mtrfr*$^{+/-}$, and asterisks indicate termination.

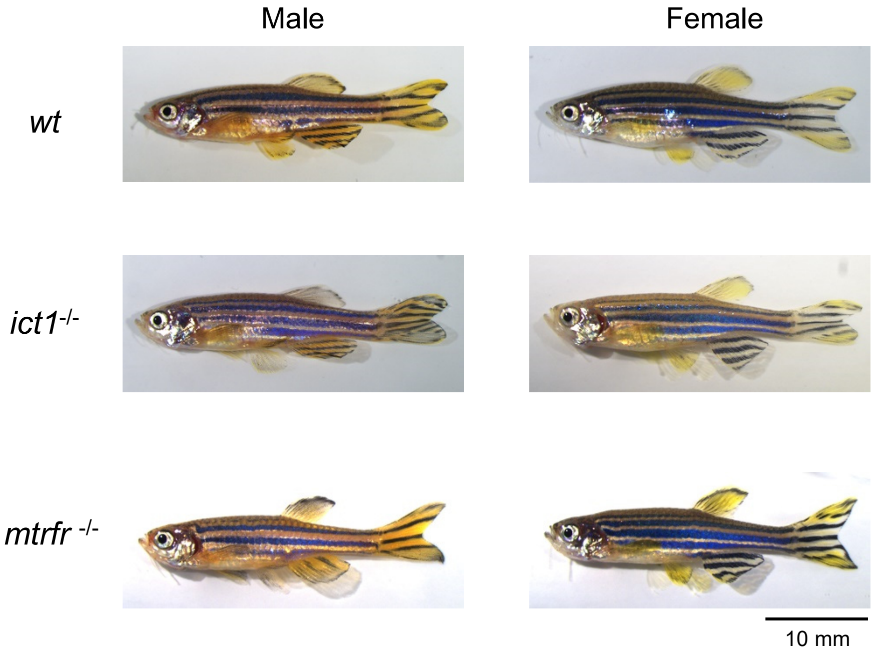

Fig. S2. Representative brightfield images of adult male and female zebrafish of the wild-type, *ict1*$^{-/-}$, and *mtrfr*$^{-/-}$ lines.

No apparent differences in phenotype were found between the wild-type and the two knockout lines.

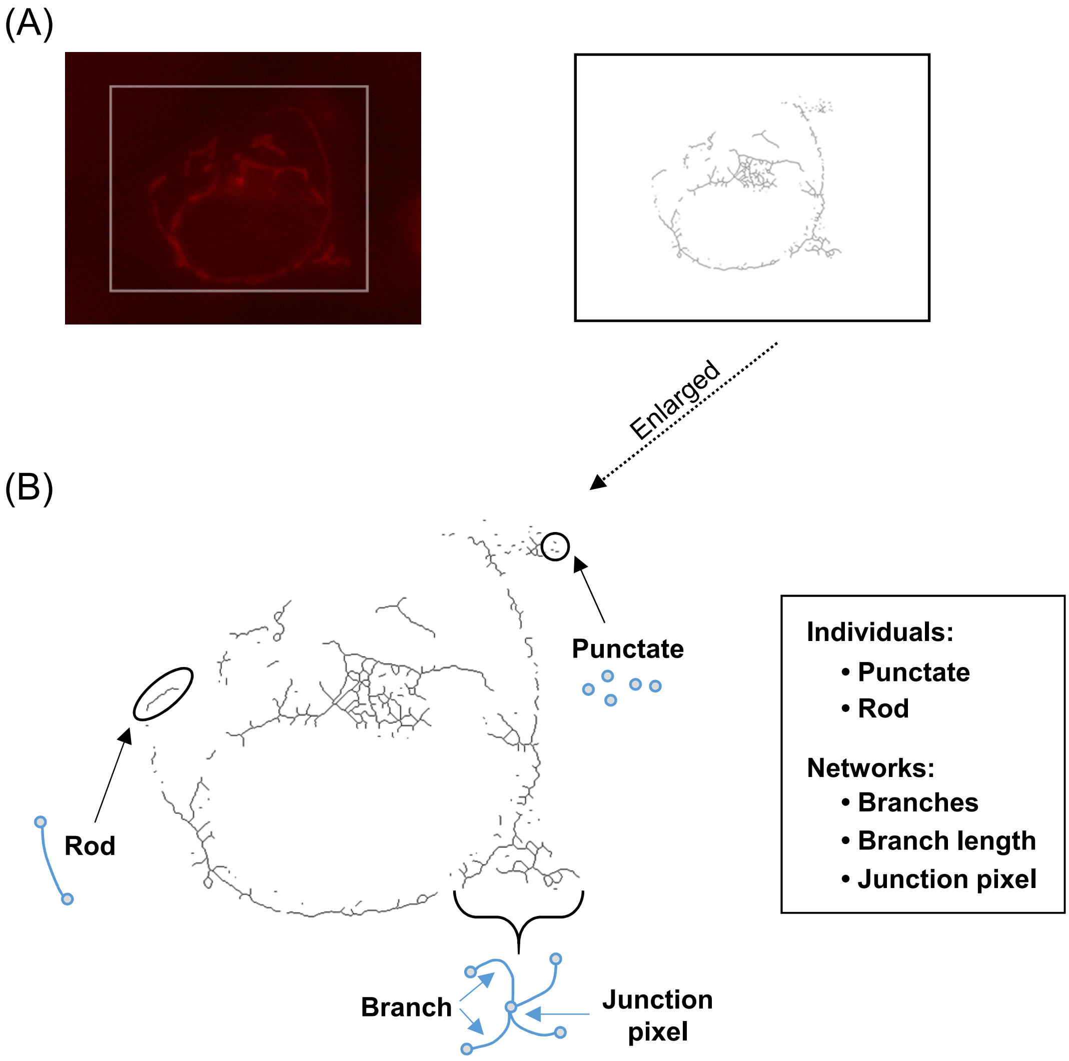

Fig. S3. Example of mitochondrial features recognized using the MiNA software.
(A) A representative fluorescent image of the caudal fin cells from adult fish of the wild-type line stained with MitoTracker Red CMXRos (*left*) and the corresponding skeletonized image created using the MiNA software (*right*), identical to the images in the upper left side of Fig. 2A. (B) Enlarged image of the skeletonized image showing mitochondrial features recognized using the MiNA software. MiNA recognizes only two types of mitochondrial structures: individuals and networks. The figure illustrates the structures and their nomenclature included in these two types.

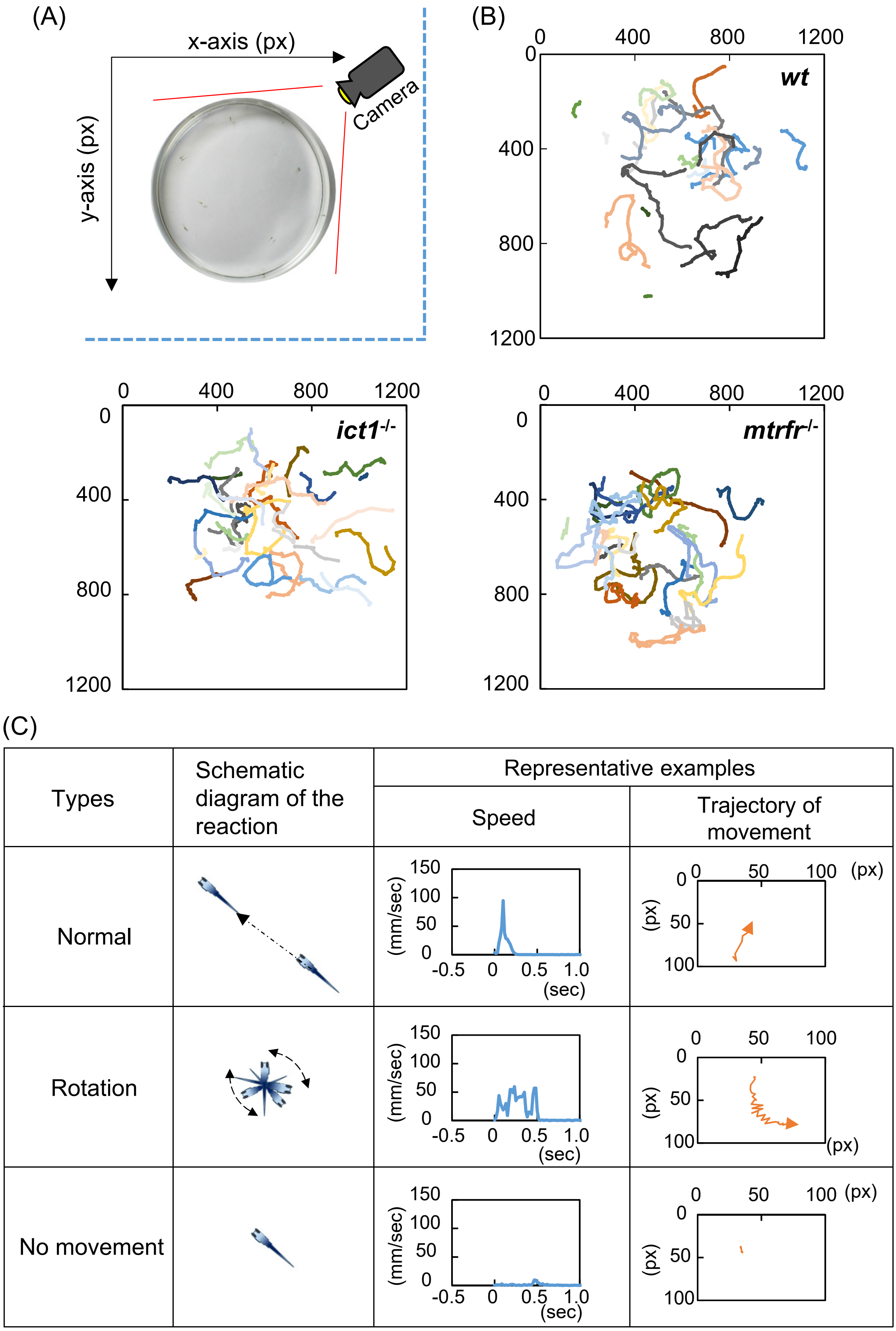

Fig. S4 (legend on next page)

Fig. S4. Behavioral assays for the larvae of the wild type, *ict1*$^{-/-}$, and *mtrfr*$^{-/-}$.

(A) Schematic diagram for observing the movement of the larvae. Ten larvae were placed in a Petri dish, and the movements of all larvae were photographed simultaneously with a digital camera. (B) Swimming trajectories of the larvae of the wild type (n = 29), *ict1*$^{-/-}$ (30), and *mtrfr*$^{-/-}$ (24) for approximately 30 s. The heads of selected larvae were marked, and their trajectories were color-coded. The results of the analysis of these data are shown in Fig. 4B. (C) The three types of touch responses to the larvae. Touching the dorsal tail region with an eyelash probe began at 0 sec.

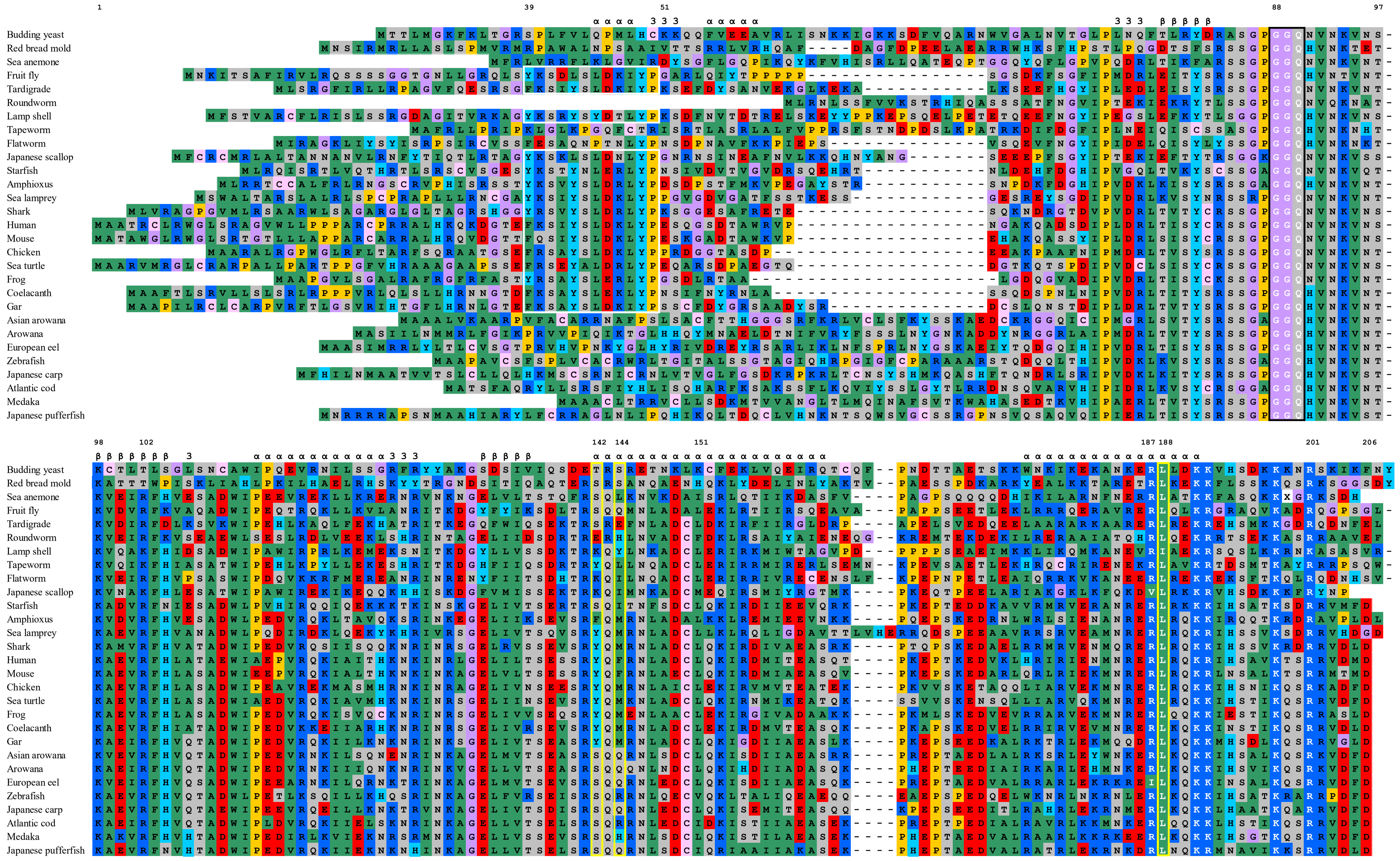

Fig. S5 (legend on next page)

Fig. S5. Sequence alignment of ICT1 proteins among a diverse range of eukaryotes. Residue numbers above the alignment indicate those in the human ICT1 protein. Secondary structure elements are indicated above the alignment according to the human ICT1 structures (PDB ID 1J26 and 7QI4). According to the structure, residues that appear to interact with ribosomal proteins are indicated by yellow boxes (refer to Supplementary Fig. 6). The YSLDK motif is indicated in a white box. The GGQ motif is shown in white and boxed in black. Residues in the C-terminal extension that interact with residues in the mRNA entry channel of the ribosome are shown in white [8]. The alignments were conducted using the ClustalW program [58], with some manual adjustments made where necessary. Alignments are colored as follows: purple: glycine (G); yellow: proline (P); green: small and hydrophobic amino acids (A, V, L, I, M); pink: hydrophobic aromatic residues (F, W); gray: hydroxyl and amine amino acids (S, T, N, Q); red: negatively charged amino acids (D, E); blue: positively charged amino acids (K, R); pale pink: cysteine (C); cyan: histidine (H) and tyrosine (Y). Accession codes used in the sequence alignment of ICT1 proteins were as follows: Budding yeast (*Saccharomyces cerevisiae*, ONH79993.1), Red bread mold (*Neurospora crassa* OR74A, XP_011395130.1), Sea anemone (*Exaiptasia diaphana*, KXJ22734.1), Fruit fly (*Drosophila melanogaster*, NP_609416.1), Tardigrade (*Hypsibius exemplaris*, OQV14855.1), Roundworm (*Caenorhabditis elegans*, NP_498174.1), Lamp shell (*Lingula anatine*, XP_013394428.1), Tapeworm (*Echinococcus granulosus*, KAH9277715.1), Flatworm (*Schistosoma japonicum*, KAH8857615.1), Japanese scallop (*Mizuhopecten yessoensis*, OWF49789.1), Starfish (*Asterias rubens*, XP_033631007.1), Amphioxus (*Branchiostoma belcheri*, XP_019615637.1), Sea lamprey (*Petromyzon marinus*, XP_032832023.1), Shark (*Callorhinchus milii*, XP_007886949.2), Human (*Homo sapiens*, NP_001536.1), Mouse (*Mus musculus*, AAH28523.1), Chicken (*Gallus*, XP_420117.3), Sea turtle (*Chelonia mydas*, XP_007065098.2), Frog (*Xenopus tropicalis*, XP_002940027.3), Coelacanth (*Latimeria chalumnae*, XP_005998315.1), Gar (*Lepisosteus oculatus*, XP_015211437.1), Asian arowana (*Scleropages formosus*, XP_018617204.1), Arowana (*Brienomyrus longianalis*, XP_048871748.1), European eel (*Anguilla*, XP_035259040.1), Zebrafish (*Danio rerio*, NP_001313644.1), Japanese carp (*Cyprinus carpio*, XP_042590815.1), Atlantic cod (*Gadus morhua*, XP_030195593.1), Medaka (*Oryzias latipes*, XP_004086834.1), and Japanese pufferfish (*Takifugu rubripes*, XP_011616819.1).

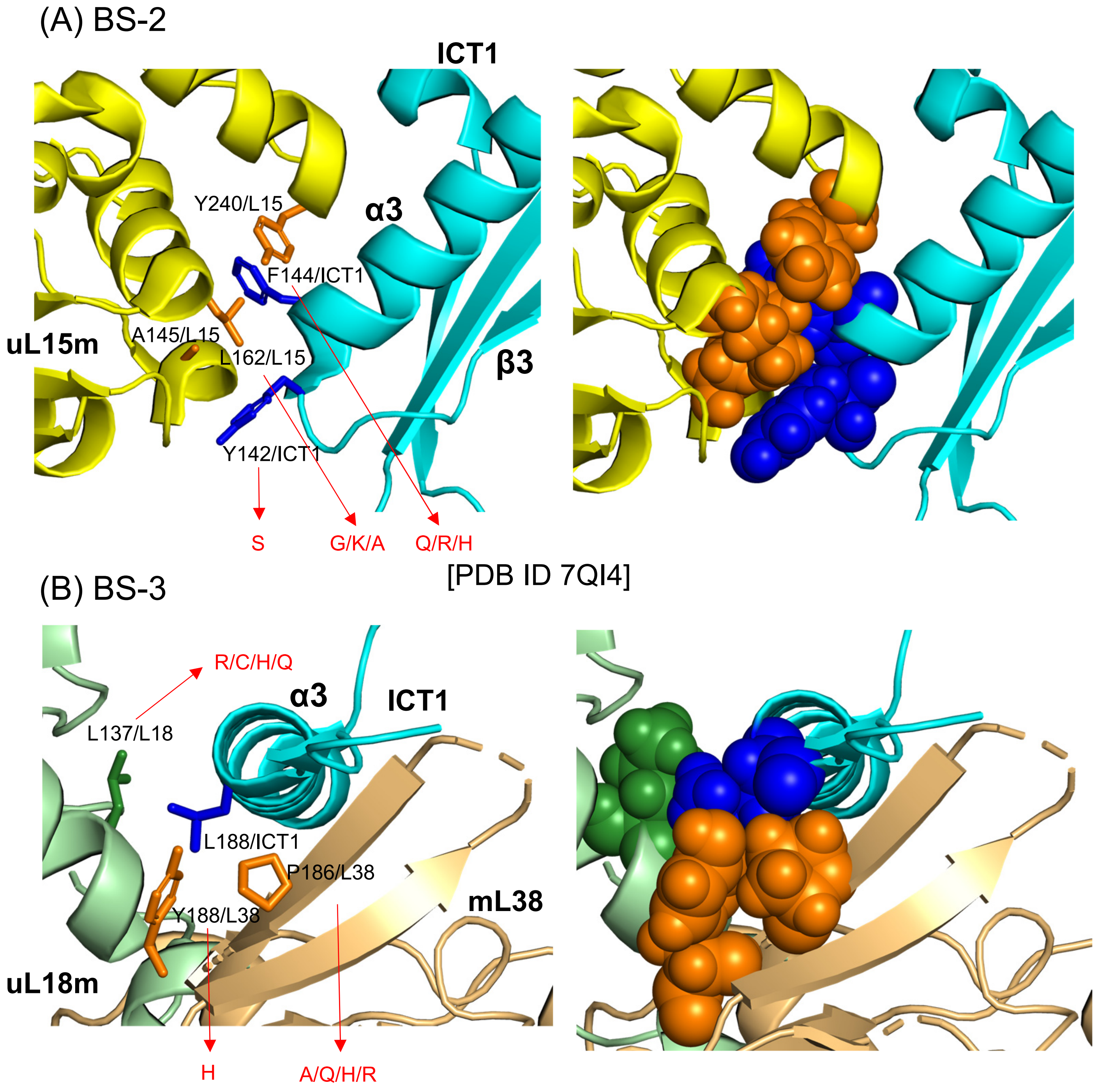

Fig. S6. Hydrophobic interactions between ICT1 and ribosomal proteins regarding BS-2 and BS-3.

(A) *Left*: BS-2 of human ICT1 (aquamarine) to uL15m (yellow) on the ribbon diagram. The structures in this figure are all based on PDB ID 7QI4 [44]. Side chains of residues involved in a hydrophobic core of interaction are shown. Residues marked by red arrows indicate those at the corresponding positions in teleosts (Supplementary Fig. 7). The program PyMOL was used to visualize the structures. *Right*: Residues involved in the hydrophobic core are presented in a CPK model. (B) BS-3 of human ICT1 (aquamarine) to uL18m (forest) and mL38 (brown) on the ribbon diagram. The others are the same as in (A).

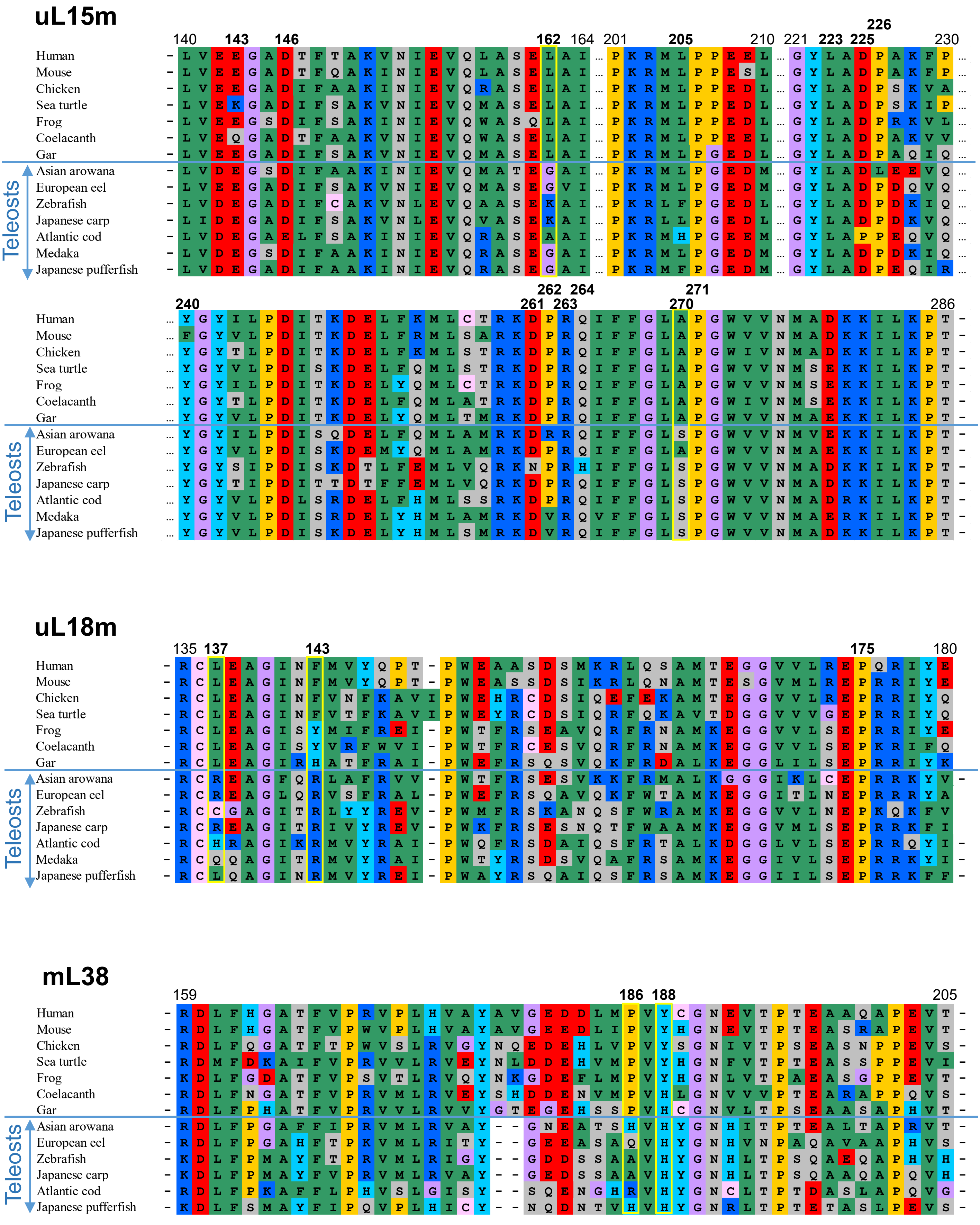

Supplementary Fig. 7 (legend on next page)

Fig. S7. Sequence alignment of uL15m, uL18m, and mL38 among metazoans.

The alignments were performed using the ClustalW program [58]. Those regions of the alignments related to BS-1, -2, and -3 are only shown; the numbers indicate the residue numbers in human ICT1. Numbers in bold indicate residues that interact with those in human ICT1. Positions of residues indicated by the red arrows in Supplementary Fig. 6 are enclosed by yellow squares; residues of each position differ between teleosts and the others. Colored indications are the same as in Supplementary Fig. 5.

Accession codes used in the uL15m sequence alignment were as follows: Human (*H. sapiens*, Q9P015.1), Mouse (*M. musculus*, Q9CPR5.1), Chicken (*G. gallus*, NP_001006388.2), Sea turtle (*C. mydas*, XP_037748541.1), Frog (*X. tropicalis*, NP_001120107.1), Coelacanth (*L. chalumnae*, XP_005993499.1), Gar (*L. oculatus*, XP_015209152.1), Asian arowana (*S. formosus*, XP_018603534.1), European eel (*A. anguilla*, XP_035269757.1), Zebrafish (*D. rerio*, NP_001003435.1), Japanese carp (*C. carpio*, XP_042584345.1), Atlantic cod (*G. morhua*, XP_030204723.1), Medaka (*O. latipes*, XP_004081189.1), and Japanese pufferfish (*T. rubripes*, XP_003968127.1).

Accession codes used in the uL18m sequence alignment were as follows: Human (*H. sapiens*, NP_054880.2), Mouse (*M. musculus*, NP_080586.1), Chicken (*G. gallus*, XP_040523889.1), Sea turtle (*C. mydas*, XP_007065747.1), Frog (*X. tropicalis*, NP_989288.1), Coelacanth (*L. chalumnae*, XP_005999322.1), Gar (*L. oculatus*, XP_006634214.2), Asian arowana (*S. formosus*, XP_018610473.1), European eel (*A. anguilla*, XP_035274340.1), Zebrafish (*D. rerio*, NP_001070798.1), Japanese carp (*C. carpio*, XP_018922493.1), Atlantic cod (*G. morhua*, XP_030223404.1), Medaka (*O. latipes*, XP_004073346.1), and Japanese pufferfish (*T. rubripes*, XP_003970431.1).

Accession codes used in the mL38 sequence alignment were as follows: Human (*H. sapiens*, NP_115867.2), Mouse (*M. musculus*, NP_077139.2), Chicken (*G. gallus*, NP_001108101.2), Sea turtle (*C. mydas*, XP_037770184.1), Frog (*X. tropicalis*, NP_001017146.1), Coelacanth (*L. chalumnae*, XP_014353460.2), Gar (*L. oculatus*, XP_006635168.2), Asian arowana (*S. formosus*, XP_018617123.2), European eel (*A. anguilla*, XP_035260903.1), Zebrafish (*D. rerio*, NP_998110.1), Japanese carp (*C. carpio*, XP_042591938.1), Atlantic cod (*G. morhua*, XP_030195300.1), and Japanese pufferfish (*T. rubripes*, XP_003977421.1).

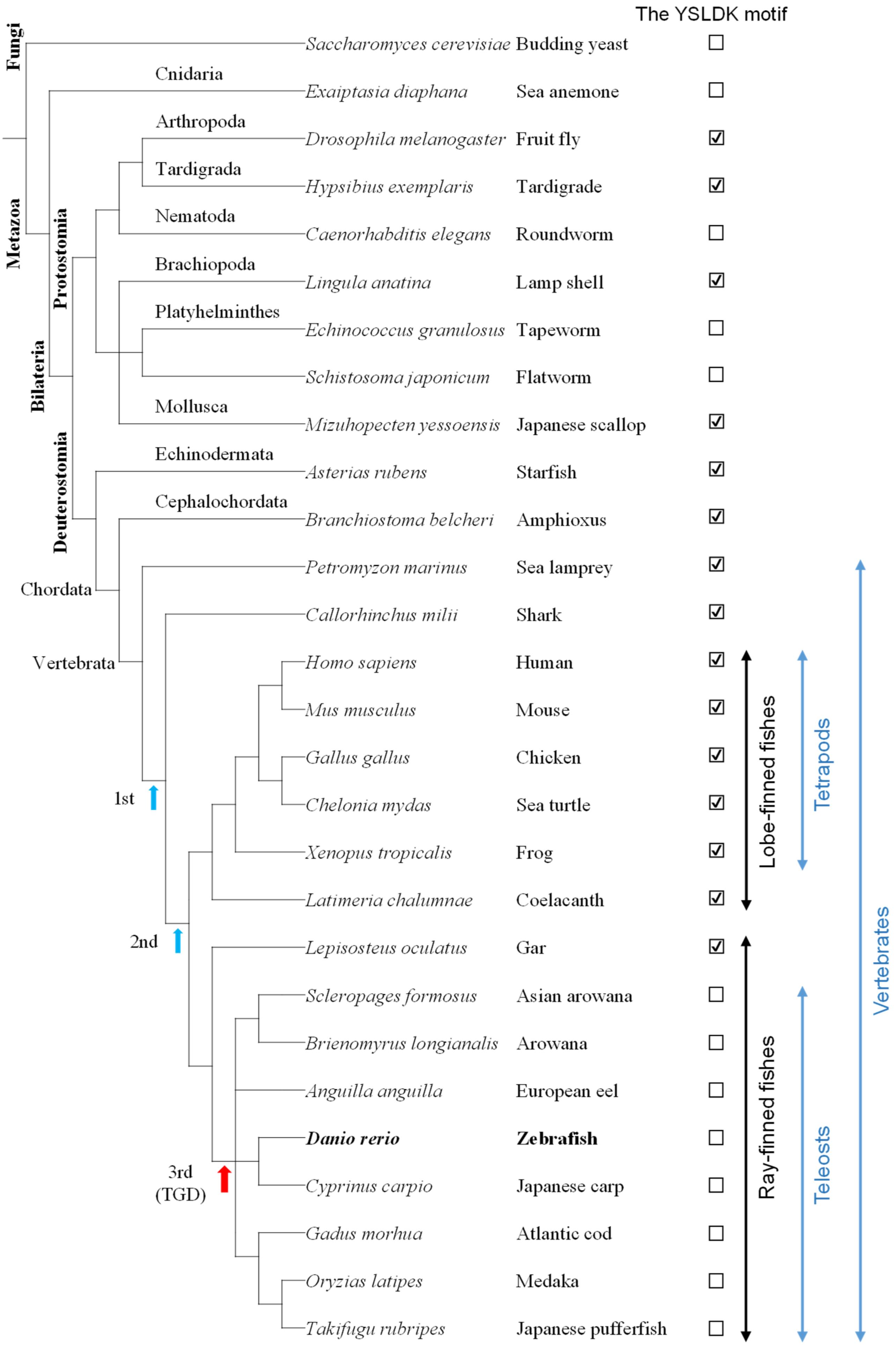

Fig. S8 (legend on next page)

Fig. S8. Simplified phylogeny tree of metazoans indicating the presence of the YSLDK motif.

The phylogenetic tree was prepared as previously described [60,61]. Teleosts (the infraclass Teleostei) account for half of all extant vertebrates and approximately 98% of all ray-finned fish species (the class Actinopterygii) [56]. A checkmark in the box indicates the presence of the YSLDK motif in the ICT1 protein of the specified species. ICT1 from the metazoan ancestor seems to have had the property of being a mitoribosomal protein. Based on the presence or absence of the YSLDK motif, we speculate that in Deuterostomia, ICT1 proteins have retained their mitoribosomal property during evolution, whereas in Protostomia, ICT1 proteins from some species have lost their mitoribosomal nature.

60 Telford MJ and Copley RR (2011) Improving animal phylogenies with genomic data. *Trends Genet* **27**, 186-195.

61 Edgecombe GD, Giribet G, Dunn CW, Hejnol A, Kristensen RM, Neves RC, Rouse GW, Worsaae K and Sørensen MV (2011) Higher-level metazoan relationships: recent progress and remaining questions. *Org Divers Evol* **11**, 151-172.

Table S1. Primers used for PCR experiments to confirm the genotypes of mice or zebrafish in this study.

**Name**	**Sequence (5′ → 3′)**	**Purpose**
Target 12 aa (F)	CCAGGGTGTCATCTTGAGAGC	Check of the genotypes of *Mtrfr* knockout mice
Target 12 aa (R)	GGAGAAGCCAGCACTGCTCT	
ict1-F	TTGAAGTAACCGCCCATTCGC	Check of the genotypes of *ict1* knockout zebrafish
ict1-R	AATGCAGTCATTAATAATCAGC	
c12orf65-F	CTGCTGGACACTGAAATATG	Check of the genotypes of *mtrfr* knockout zebrafish
c12orf65-R	TCTTAAATATTATTAAGGAG	

Table S2. Summary of the symbols of the genes and proteins for the two ribosome rescue factors in different eukaryotes.

Species	Rescue factor 1	Rescue factor 2
H. sapiens	*ICT1* / ICT1<br>*MRPL58* / MRPL58, mL62	*MTRFR* / mtRF-R, MTRFR<br>*C12orf65* / C12orf65
M. musculus	*Ict1* / ICT1	*Mtrfr* / MTRFR
D. rerio	*ict1* / Ict1	*mtrfr* / Mtrfr
S. cerevisiae	*PTH4* / Pth4	*PTH3* / Pth3
